# Exploring tunneling ESEEM beyond methyl groups in nitroxides at low temperatures†

**DOI:** 10.1039/d4cp01212g

**Published:** 2024-05-07

**Authors:** Andrea Eggeling, Thacien Ngendahimana, Gunnar Jeschke, Gareth R. Eaton, Sandra S. Eaton

**Affiliations:** a ETH Zurich, Department of Chemistry and Applied Biosciences Vladimir-Prelog-Weg 2 8093 Zurich Switzerland gjeschke@ethz.ch; b Department of Chemistry and Biochemistry, University of Denver Denver CO 80208 USA sandra.eaton@du.edu

## Abstract

Tunneling of methyl rotors coupled to an electron spin causes magnetic field independent electron spin echo envelope modulation (ESEEM) at low temperatures. For nitroxides containing alkyl substituents, we observe this effect as a contribution at the beginning of the Hahn echo decay signal occurring on a faster time scale than the matrix-induced decoherence. The tunneling ESEEM contribution includes information on the local environment of the methyl rotors, which manifests as a distribution of rotation barriers *P*(*V*_3_) when measuring the paramagnetic species in a glassy matrix. Here, we investigate the differences in tunneling behaviour of geminal methyl and ethyl group rotors in nitroxides while exploring different levels of theory in our previously introduced methyl quantum rotor (MQR) model. Moreover, we extend the MQR model to analyze the tunneling ESEEM originating from two different rotor types coupled to the same electron spin. We find that ethyl groups in nitroxides give rise to stronger tunneling ESEEM contributions than methyl groups because the difference between hyperfine couplings of their methyl protons better matches the tunneling frequency. The methyl rotors of both ethyl and propyl groups exhibit distributions at lower rotation barriers compared to geminal methyl groups. This is in good agreement with density functional theory (DFT) calculations of their rotation barriers and showcases that conformational flexibility impacts the hindrance of rotation. Using Monte-Carlo based fitting in combination with an identifiability analysis of the MQR model parameter space, we extract rotation barrier distributions for the individual rotor types in mixed-rotor nitroxides as well as identify which rotors dominate the observed tunneling contribution in the Hahn echo decay signal.

## Abstract 2

The conformational flexibility of ethyl groups manifests in their methyl rotor tunnelling behaviour observed with ESEEM spectroscopy at low temperatures resulting in lower rotation barriers for ethyl groups than for methyl groups in nitroxides.

## Graphical abstract

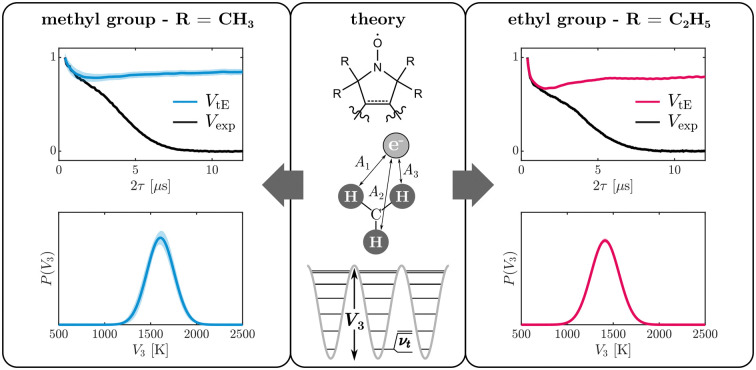

## 1 Introduction

Nitroxides are omnipresent paramagnetic molecules in electron paramagnetic resonance (EPR) spectroscopy as spin labels^1–3^ and polarising agents^4–6^ as well as model systems in method development^7–9^ and investigation of fundamental spin dynamics.^10^ Understanding their relaxation behaviour is of great importance since the electron spin decoherence determines the accessible distance range with dipolar EPR techniques^11,12^ and impacts the polarization transfer in dynamic nuclear polarization (DNP) experiments.^13^ Generally, electron spin decoherence is influenced by several parameters including molecular structure,^14–16^ matrix composition,^17–19^ methyl groups^20–24^ and temperature,^21,24,25^ which have been the focus of extensive studies in the last decades. In the low-temperature regime, nitroxides with geminal methyl groups exhibit two contributions to the Hahn echo decay on characteristic time scales.^21,23^ Nuclear pair electron spin echo envelope modulation (ESEEM) causes the slower matrix-driven decoherence contribution,^26^ which can be modelled quantitatively using cluster correlation expansion (CCE).^19,26–29^ The faster contribution has only been attributed recently to methyl tunneling ESEEM with the help of regularized noise spectroscopy,^18^ even though a theoretical study predicted this quantum mechanical phenomenon already 20 years ago.^30^

Methyl group rotation occurs within a threefold potential with a characteristic rotation barrier *V*_3_ due to intra- and intermolecular interactions between the rotor and its chemical environment.^31,32^ In other words, the rotation barrier *V*_3_ is sensitive to local hindrance around the methyl rotor.^31^ The tunneling frequency *ν*_t_, which corresponds to the splitting of the energy levels within each ro-librational state *r*, is closely related to *V*_3_ and depends on the wavefunction overlap between the potential wells.^33^ Higher rotation barriers lead to less wavefunction overlap which results in smaller tunneling frequencies.^32^ At low temperatures, quantum rotational tunneling becomes the dominant proton position exchange process for methyl rotors. ESEEM pulse sequences coherently manipulate the electron spin and thereby generate formally forbidden coherences on the tunneling states (A, E_a_, E_b_) of the ro-librational ground state *r* = 0.^33^ Therefore, the Hahn echo produced by the two-pulse ESEEM pulse sequence is modulated with the tunneling frequency *ν*_t_ if the quantum rotor is coupled to the manipulated and observed electron spin.^24,33,34^ In the tunneling temperature regime, nitroxides are usually measured in a glassy matrix where the local environment influencing the rotation barrier *V*_3_ is not uniform. A distribution of rotation barriers *P*(*V*_3_) must be considered to account for the variety of local hindrance experienced by the nitroxides' methyl rotors. The methyl quantum rotor (MQR) model integrates the spin dynamics of the tunneling ESEEM contribution and the nuclear pair ESEEM contribution, to determine the underlying rotation barrier distribution *P*(*V*_3_) in a glassy matrix.^24^ Since the tunneling ESEEM phenomenon is not limited to methyl group rotors and/or nitroxide spin systems,^33,35^ different quantum rotors can also be experimentally investigated with ESEEM spectroscopy and analyzed with the MQR model.

Conventional spin probes and labels feature two pairs of geminal methyl groups close to the nitroxide functionality. Substitution of these methyl groups by ethyl groups enhances stability of the nitroxides in reducing environments,^36^ which is of interest for in cell EPR studies.^37^ Further, it is known that thermally activated rotation of methyl groups close to a paramagnetic center causes an increase in the transverse relaxation rate at temperatures above about 60 K.^14,17,20,38^ As one expects this effect to decrease with increasing distance of the methyl groups from the paramagnetic center, substitution of the pairs of geminal methyl groups by pairs of ethyl groups is expected to reduce transverse relaxation in this temperature range. For the application of such spin labels in pulse EPR experiments, it is of interest to study the tunneling ESEEM contribution to electron spin echo decay in the low-temperature regime.

Here, we aim to characterize the rotation barrier distribution of methyl rotors in various alkyl groups in nitroxides using the MQR model^24^ and thereby gain insight into the near-range environment of different nitroxides investigated in a glassy matrix. The systematic study of methyl rotors in different alkyl groups of nitroxides allows us to identify how sensitive the tunnelling ESEEM contribution is towards changes in the rotation barrier, and if we can identify these differences with our proposed model and fitting approach. In Section 2, we introduce the Hamiltonian to describe the tunneling phenomenon, its approximations and how the spin dynamics translate to the tunneling ESEEM contribution in the MQR model. Section 3 presents the investigated model compounds, the experimental set-up, the fitting procedure to infer the rotation barrier distribution as well as computational efforts to characterize the rotation barrier(s) of the investigated spin systems. In Section 4, we evaluate what Hamiltonian is required to represent the experimental tunneling ESEEM contribution in the Hahn echo decay signal. Moreover, we summarize how to treat equivalent- and mixed-rotor nitroxides with the MQR model and interpret the evaluated rotation barrier distributions for the different spin systems. At the end, we conclude by summarizing the differences in tunneling ESEEM and rotation barrier distribution of the investigated methyl rotors in various alkyl groups and the insights gained for mixed-rotor nitroxides.

## 2 Theory

### 2.1 Tunneling ESEEM of methyl rotors in alkyl groups

The Hamiltonian describing the tunneling ESEEM phenomenon is separable into a spin and a tunneling Hamiltonian, respectively. The methyl group rotation angle *ϕ* determines the spin Hamiltonian^23,24,33^ for each localized state of the rotor according to

1 *Ĥ*(*ϕ*) = *Ĥ*_Z_ + *A*_I_(*ϕ*)*Ŝ*_*z*_*Î*_1,*z*_ + *A*_II_(*ϕ*)*Ŝ*_*z*_*Î*_2,*z*_ + *A*_III_(*ϕ*)*Ŝ*_*z*_*Î*_3,*z*_ + *B*_I_(*ϕ*)*Ŝ*_*z*_*Î*_1,*z*_ + *B*_II_(*ϕ*)*Ŝ*_*z*_*Î*_2,*z*_ + *B*_III_(*ϕ*)*Ŝ*_*z*_*Î*_3,*z*_

where *Ĥ*_Z_ includes the electron and nuclear Zeeman terms, *A*_*i*_(*ϕ*) represents the secular and *B*_*i*_(*ϕ*) the pseudo-secular hyperfine coupling constants for the methyl proton position *i*, respectively. The secular hyperfine coupling constant *A*_*i*_(*ϕ*) consists of two contributions. The first contribution is isotropic and originates from the so-called Fermi contact interaction *A*_*i*,FC_ which accounts for spin density of the unpaired electron in the atomic s-orbital of the coupled nucleus. The second contribution is the dipolar interaction *A*_*i*,DD_, which depends on the distance *r*_*i*_ between the two coupling partners and their gyromagnetic ratios *γ*_e_ and *γ*_H_. The pseudo-secular hyperfine coupling *B*_*i*_(*ϕ*) is also part of the dipolar interaction.2
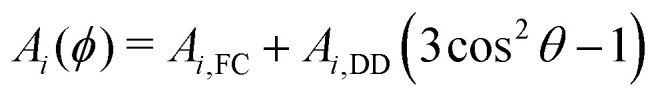

3 *B*_*i*_(*ϕ*) = 3*A*_*i*,DD_ sin *θ* cos *θ*

4
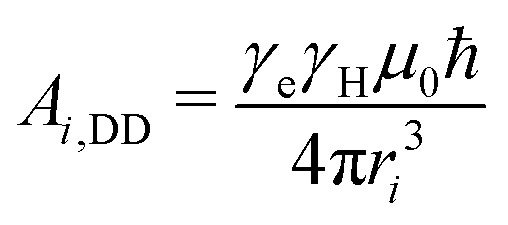
Since the dipolar interaction is anisotropic, the secular and pseudo-secular hyperfine constants both depend on the orientation *θ* of the interspin vector between the coupled electron spin and rotor proton with respect to the external magnetic field. The Fermi contact as well as the dipolar interaction are closely related to the molecular structure and conformation of the methyl rotor containing paramagnetic molecule. This indicates that the spin Hamiltonian is different for rotors on methyl and ethyl substituents in common nitroxides used for site-directed spin labelling of proteins.^1,39^

The tunneling Hamiltonian based on the hindered rotor model^32^ describes the methyl group rotation potential by the tunneling frequency *ν*_t_5
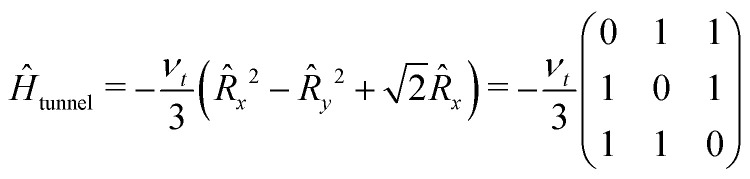
where *R* represents a pseudo-spin with spin *R* = 1.^24^ The rotation barrier is sensitive to local hindrance and therefore its local environment meaning intra- and intermolecular interactions in the rotor's proximity.^40^ Therefore, the rotation barrier as well as the tunneling Hamiltonian differ for different types of rotors, like for the previously mentioned geminal methyl and ethyl substituents in nitroxide spin labels.

The full state Hamiltonian *Ĥ*_free_ consists of the spin Hamiltonian for the localized rotor states on the diagonal, which are mixed by the off-diagonal terms of the tunneling Hamiltonian.^24,33^ Due to the differences in both the spin and tunneling Hamiltonian, we observe different tunneling ESEEM contributions to the Hahn echo decay for nitroxides containing different types of alkyl groups.

ESEEM spectroscopy probes methyl tunneling in the low-temperature regime where it is the dominant methyl proton exchange process. In this temperature regime, we investigate the paramagnetic molecule in a glassy matrix consisting of a solvent or solvent mixture that forms a glass when cooled. This leads to varying local environments around the methyl rotors influencing their rotation barrier *V*_3_. The methyl quantum rotor (MQR) model accounts for this by a distribution of rotation barriers.^24^ The tunneling ESEEM contribution to the overall Hahn echo decay signal is expressed as6
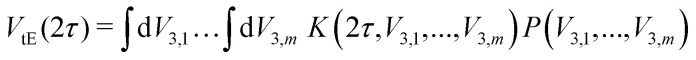
where *K* is the tunneling ESEEM kernel and *P*(*V*_3,1_, *V*_3,2_,…, *V*_3,*m*_) the multi-variate rotation barrier distribution accounting for the local environment of the *m* different methyl rotor types *i* = 1,…, *m*. The tunneling ESEEM kernel considers the full state Hamiltonian of the coupled methyl rotors to the investigated electron spin under application of the pulse sequence^24^ to represent the spin dynamics. The experimentally observed Hahn echo decay, *i.e.* two-pulse ESEEM, signal is modelled as the product of the tunneling ESEEM contribution *V*_tE_ and a background function *V*_bg_ according to7
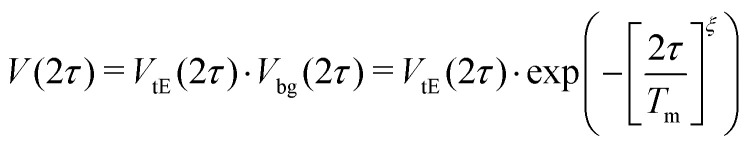
where *V*_bg_ accounts for the matrix-induced decoherence process by a stretched exponential characterized by the phase memory *T*_m_ and the stretch parameter *ξ*. Nuclear-pair ESEEM due to nitroxide backbone protons proceeds on the same slow time scale as the one due to matrix protons and is thus also accounted for by *V*_bg_.

### 2.2 Tunneling ESEEM matching condition

The difference in hyperfine (HF) coupling constants as well as rotation barriers for the methyl rotors of methyl, ethyl and longer alkyl substituents impacts their experimentally observed tunneling ESEEM contribution to the Hahn echo decay. This is due to the fact that tunneling ESEEM requires matching of the differences in HF constants at the proton positions with the tunneling frequency,^33^ which are both molecule-dependent properties. To determine this matching condition, we calculated the difference in secular hyperfine coupling constants for 841 orientations of the rotor with respect to the external magnetic field. We used DFT to calculate the Fermi contact and dipolar HF contribution as well as the constrained rotation barrier *V*^c^_3_ in vacuum (see Section 3.2). The tunneling frequency *ν*_t_ was obtained from the rotation barrier using the hindered rotor model.^32^ We used density operator formalism to simulate single-orientation tunneling ESEEM signals for methyl rotors in different alkyl groups to evaluate the tunneling ESEEM modulation depth according to8
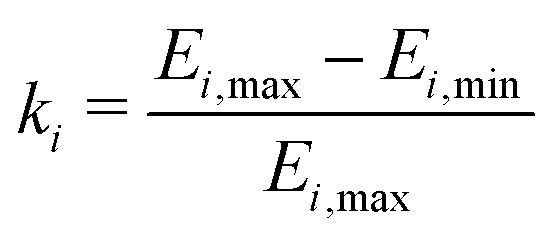
where *E*_*i*_ represents the single-orientation tunneling ESEEM signal of orientation *i*.

**Fig. 1:**
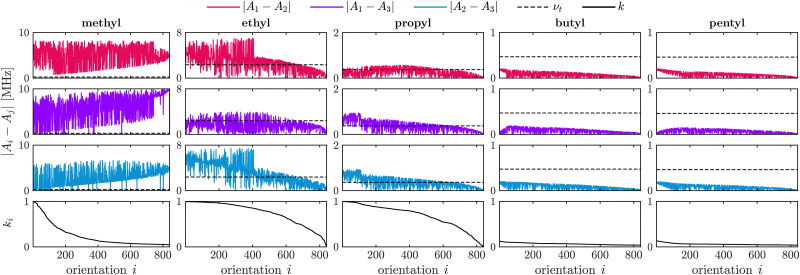
Simulations showing the relation between hyperfine coupling constants, tunneling frequency and tunneling ESEEM modulation depth for methyl rotors of different alkyl substituents. The parameters influencing the tunneling ESEEM matching condition were evaluated using the DFT-calculations for the rotors of the methyl, ethyl, propyl, butyl and pentyl substituents (*m* = 3) in 2-DOXYL-C_11_, 3-DOXYL-C_14_, 4-DOXYL-C_14_, 5-DOXYL-C_14_ and 6-DOXYL-C_14_, respectively. The differences in hyperfine constants |*A*_*i*_ − *A*_*j*_| are sorted for decreasing tunneling modulation depth *k*_*i*_ in the simulated single-orientation two-pulse ESEEM signal. The orientation number *i* indexes the orientations on a spherical grid used to calculate |*A*_*i*_ − *A*_*j*_| and simulate single-orientation tunneling ESEEM signals. The tunneling frequency *ν*_t_ evaluated from the DFT-calculated constrained surface scan rotation barrier *V*^c^_3_ is given as a dashed black line. The apparent noise for the calculated differences in hyperfine coupling constants |*A*_*i*_ − *A*_*j*_| comes from sorting the orientations *i* according to decreasing tunneling ESEEM modulation depth.

Fig. 1 illustrates the relation between the differences in secular HF constants |*A*_*i*_ − *A*_*j*_|, the tunneling frequency *ν*_t_ and the two-pulse ESEEM modulation depth *k* for different alkyl substituents in nitroxides. The alkyl chain length containing the investigated rotor increases from a methyl to a pentyl substituent. The difference in HF coupling constants decreases for longer alkyl chains from left to right in Fig. 1 due to less spin density of the electron spin at the rotor protons leading to smaller Fermi contact contributions as well as smaller dipolar interaction because of the inversely proportional distance dependence (see eqn (2)). The tunneling frequency increases from a methyl rotor in geminal methyl groups (*ν*_t_ = 231 kHz) to the one in an ethyl group (*ν*_t_ = 2.36 MHz), then decreases and remains almost constant for longer alkyl chains like propyl (*ν*_t_ = 365 kHz), butyl (*ν*_t_ = 471 kHz) and pentyl groups (*ν*_t_ = 463 kHz). This leads to very good matching between the differences of HF coupling constants with the tunneling frequency in case of methyl rotors on ethyl and propyl groups, sufficient matching for a substantial fraction of the orientations for methyl groups, and no matching for the rotor of butyl and pentyl groups. Fig. 1 shows that efficient matching of these parameters results in large two-pulse ESEEM modulation depth *k* of the simulated single-orientation signals for the methyl rotors of methyl, ethyl and propyl groups.

Fig. 1 does not account for a distribution of rotation barriers due to different local environments experienced by the rotors in a glassy matrix. Therefore, our interpretation of the matching condition for different rotor types aims to rationalize the differences in the experimentally observed tunneling ESEEM contributions but does not fully represent the spin dynamics occurring during the Hahn echo decay measurement.

### 2.3 Impact of hyperfine terms on tunneling ESEEM

**Fig. 2:**
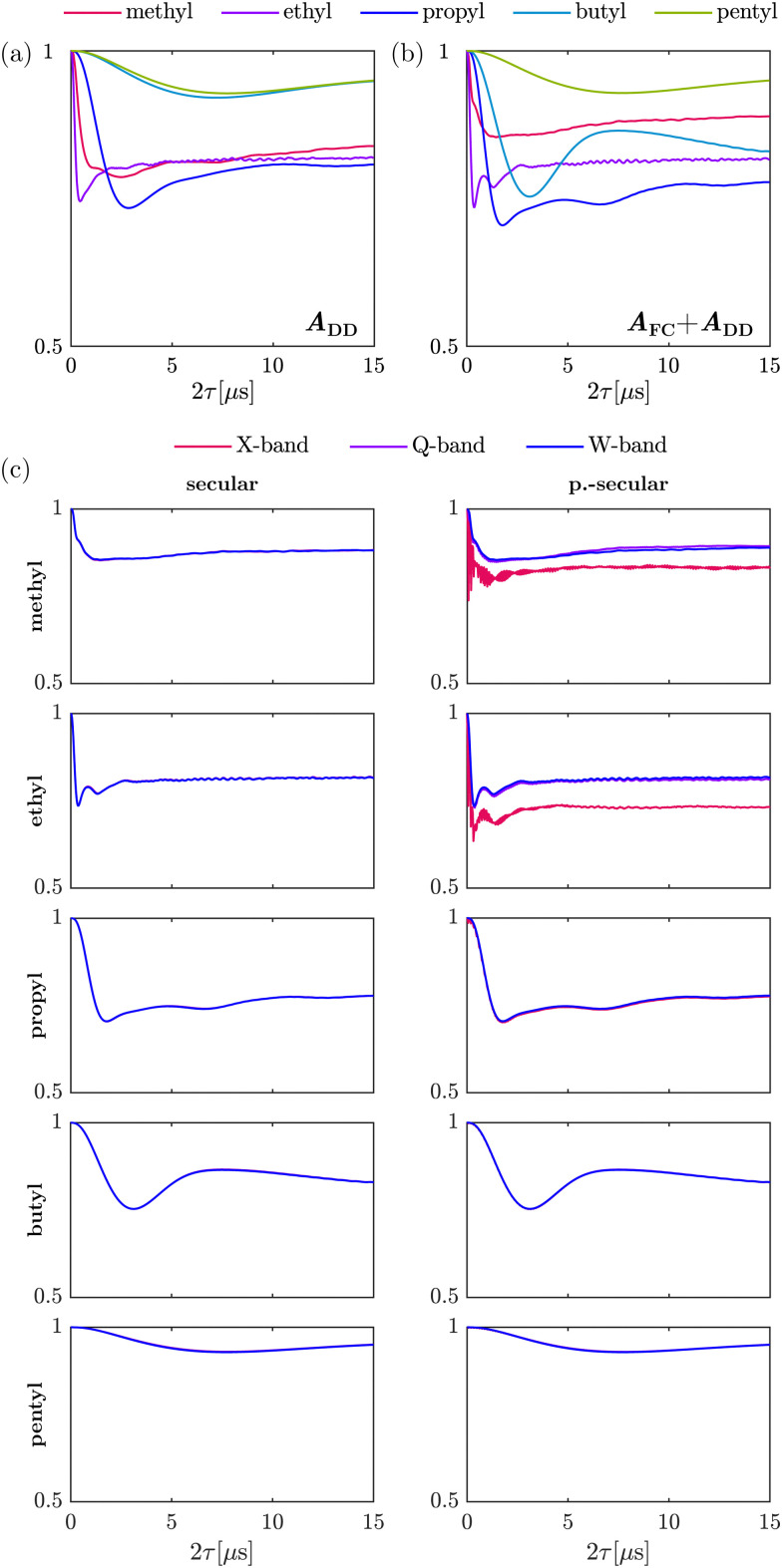
Simulations showing the influence of the Fermi contact interaction, the pseudo-secular hyperfine term and the magnetic field dependence on the tunneling ESEEM signal of methyl rotors in different alkyl groups. The tunneling ESEEM signals for the methyl rotor of methyl, ethyl, propyl, butyl and pentyl groups were simulated at W-band frequency using the DFT-calculations for rotor *m* = 3 in 2-DOXYL-C_11_, 3-DOXYL-C_14_, 4-DOXYL-C_14_, 5-DOXYL-C_14_ and 6-DOXYL-C_14_, respectively. In (a), the secular HF term consisted of the dipolar interaction ADD only, whereas in (b) the Fermi contact interaction AFC was additionally included. (c) Illustration of the influence of the pseudo-secular (p.-secular) hyperfine term as well as the magnetic field dependence of the tunneling ESEEM signal for the different alkyl rotors. All tunneling ESEEM signals were simulated with a Gaussian rotation barrier distribution with a mean value *V*_av_ corresponding to the constrained DFT-calculated rotation barrier *V*^c^_3_ and a fixed standard deviation *σ* = 200 K.

The HF interaction between the electron spin and the methyl protons relevant for tunneling ESEEM contains two terms, the secular term *A*_*i*_(*ϕ*)*Ŝ*_*z*_*Î*_*i*,*z*_ and the pseudo-secular term *B*_*i*_(*ϕ*)*Ŝ*_*z*_*Î*_*i*,*x*_. As mentioned above, the first term consists of the isotropic Fermi contact and the anisotropic dipolar contribution. Previously, for geminal methyl substituents in nitroxides the hyperfine coupling constant *A*_*i*_(*ϕ*) was approximated by the dipolar contribution only.^23,24^ In Fig. 2(a) and (b), we evaluated the influence of the Fermi contact contribution by simulating the tunneling ESEEM signal of methyl rotors in different alkyl groups for a Gaussian rotation barrier distribution using density operator formalism. The Fermi contact and dipolar HF coupling constants as well as the rotation barriers used for the simulations were evaluated using DFT-calculations (see Section 3.2).

Fig. 2(a) and (b) illustrate that the modulation depth and period of the tunneling ESEEM signal for the investigated rotors differ. In Fig. 2(a) only the dipolar HF interaction is considered. In this case, the tunneling modulation depth increases from methyl to propyl groups and starts decreasing for butyl and pentyl groups. The modulation period of the tunneling ESEEM is shortest for the methyl rotor in ethyl groups and increases for longer alkyl chains as well as for the methyl rotor in geminal methyl groups. The same trends can be observed in Fig. 2(b), where both the Fermi contact and dipolar interaction are considered in the tunneling ESEEM simulation. However, the modulation depth and period changes if the Fermi contact interaction is included for all rotors except for the case of pentyl groups. This change in modulation period and depth occurs due to a change in the difference between the proton hyperfine coupling constants when the Fermi contact interaction is considered, which impacts the tunneling ESEEM matching condition. In case of the pentyl group, the Fermi contact coupling constants are nearly zero for all rotor protons, which explains why no significant impact of this contribution can be observed. Generally, the isotropic HF interaction can impact the tunneling ESEEM signal of methyl rotors in methyl, ethyl, propyl and butyl substituents. However, in these computations we only considered a single conformation of the alkyl groups, where in reality several rotamers may have significant population leading to a distribution of Fermi contact and dipolar couplings. How significant the Fermi contact term is for the different alkyl groups in the experimentally observed tunneling ESEEM will be discussed in Section 4.1.1.

The pseudo-secular HF term needs to be considered if the high-field approximation for the coupled nuclei is not fulfilled. Well-known from nuclear ESEEM,^41^ this term allows detection of formally forbidden transitions in a coupled electron–nucleus spin pair by ESEEM spectroscopy. Nuclear ESEEM from both matrix and nitroxide protons is very significant at low magnetic fields like at X-band (9.5 GHz, 0.35 T) but becomes negligible at W-band (94 GHz, 3.35 T). The influence of the pseudo-secular term on the tunneling ESEEM at different magnetic fields is illustrated in Fig. 2(c) for the rotors in different alkyl groups.

Firstly, when only including the secular HF term, no significant differences in the simulated tunneling ESEEM signal for different magnetic fields can be observed for all alkyl substituents (see Fig. 2(c) secular). This is not surprising since there is no magnetic field dependence in the Hamiltonian when including only the *A*-term of the hyperfine coupling. Secondly, when including the pseudo-secular *B*-term in the simulation, the nuclear ESEEM of the methyl protons of methyl and ethyl substituents is clearly visible in the tunneling ESEEM signal at X-band (see Fig. 2(c) p.-secular). For longer alkyl chains, nuclear ESEEM from the methyl protons becomes negligible due to smaller dipolar couplings which leads to small *B*_*i*_(*ϕ*)-values. Thirdly, the tunneling ESEEM signals simulated with the pseudo-secular HF term for Q- and W-band are almost the same as the simulations with only the secular term for all alkyl substituents. The pseudo-secular HF term only impacts the tunneling ESEEM signal of the rotors in methyl and ethyl groups at X-band. The modulation period remains unchanged but the modulation depth increases at X-band compared to the tunneling ESEEM signals simulated for Q-band and W-band, respectively. For the methyl rotors in propyl, butyl and pentyl groups, there is no difference between the tunneling ESEEM signals at X-, Q- and W-band. Since the pseudo-secular coupling constant becomes smaller for longer alkyl chains, the *B*-term becomes irrelevant and has no influence on the tunneling ESEEM signal for these rotors. In conclusion, the pseudo-secular hyperfine term can be neglected for alkyl groups longer than ethyl groups at typical magnetic fields for X-band frequency and larger. However, for methyl and ethyl groups the pseudo-secular terms should be considered at X-band frequency and can only be omitted at Q-band and higher frequencies. In Section 4.1.1, we will discuss the relevance of the pseudo-secular HF term when inferring rotation barrier distributions from experimental ESEEM data.

## 3 Materials and methods

### 3.1 Model compounds and EPR measurements

**Fig. 3:**
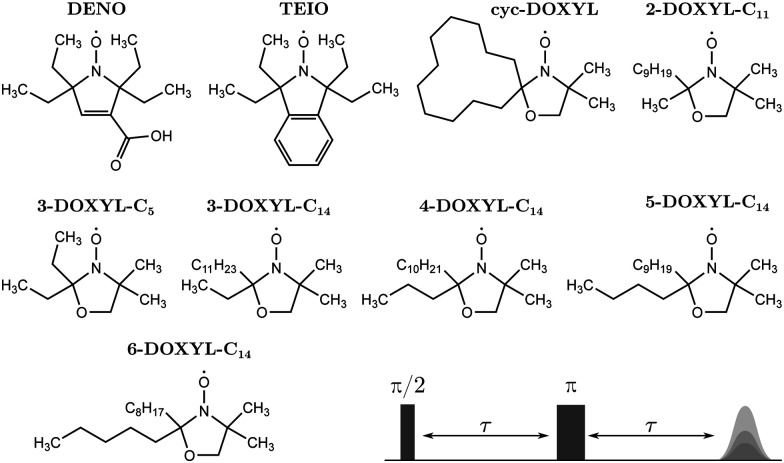
Investigated nitroxides and Hahn echo pulse sequence. Molecular structures of the pyrroline-based nitroxides containing four geminal ethyl groups *gem*-diethyl-nitroxide (DENO) and tetraethylisoindoline (TEIO) and all investigated DOXYL-based nitroxides with varying alkyl chain lengths. The variable delay Hahn echo decay, *i.e.* two-pulse ESEEM, pulse sequence is illustrated schematically.

The molecular structure of the nitroxide model compounds under investigation are illustrated in Fig. 3. DENO^42^ was a gift from Prof. Andrzej Rajca, University of Nebraska. TEIO^43^ and cyc-DOXYL^44^ were prepared as previously reported. 2-DOXYL-C_11_, 3-DOXYL-C_5_, 3-DOXYL-C_14_, 4-DOXYL-C_14_, 5-DOXYL-C_14_, and 6-DOXYL-C_14_ were prepared similarly to cyc-DOXYL using the method of Keana *et al.*^45^ Conversion of the starting ketone to the doxyl amine was monitored by IR and NMR. Oxidation of the doxyl amine to the nitroxide was monitored by quantitative EPR. The solutions for EPR spectroscopy were 0.2 to 0.3 mM in *cis*/*trans* decalin. Samples for X-band (∼9.71 GHz) were contained in 4 mm OD quartz tubes. Air was removed by several freeze–pump–thaw cycles on a vacuum line. The sample tubes were backfilled with He gas to a pressure of about 100 mtorr prior to flame sealing. The low pressure He provides thermal contact between the sample and the walls of the tube to facilitate thermal equilibration. Samples for Q-band (∼34.1 GHz) were in 1.6 mm OD quartz tubes and were not degassed. The decalin matrix was selected because it does not contain methyl groups and reliably forms a glass when cooled rapidly.

EPR measurements were performed on a Bruker E580 spectrometer using a CF935 cryostat and an ER4118XMD5 dielectric resonator at X-band or a ER5107 dielectric resonator at Q-band. Temperature was controlled with a Bruker/ColdEdge Stinger closed cycle He system. An Oxford ITC503 controller was used to control heating at the bottom of the cryostat to establish the desired temperature. Sample temperature was measured with a Lakeshore Cernox sensor located near the sample, external to the resonator. The He gas flow was fast enough that the sample temperature equilibrated with the cryostat inlet temperature in less than 15 minutes. Electron spin Hahn echo decays were measured using a π/2–*τ*–π–*τ*–echo sequence and applying a 40 ns π/2-pulse and a 80 ns π-pulse (see Fig. 3). We used such relatively long pulses to suppress proton ESEEM.^46^ Two-step phase cycling was applied to the initial π/2-pulse. The shot repetition time was adjusted to be long relative to *T*_1_.

The nitroxides 2-DOXYL-C_11_, 3-DOXYL-C_14_, 4-DOXYL-C_14_, 5-DOXYL-C_14_ and 6-DOXYL-C_14_ contain one long alkyl substituent (–C_*n*_H_2*n*+1_ with *n* ≥ 9). We neglect all methyl rotors on alkyl groups longer than pentyl groups in the analysis of the experimental Hahn echo decay data, since our investigation of the tunneling ESEEM matching condition in Section 2.2 revealed that already for butyl and pentyl groups the matching condition is not fulfilled.

### 3.2 DFT-based calculations of nitroxide properties

Simulation of the tunneling ESEEM kernel relies on several properties of the alkyl rotor containing nitroxide. We used open-shell Kohn–Sham density functional theory (DFT) in ORCA version 5.0.2^47^ to optimize the molecular geometry, evaluate rotation barriers by energy surface scans and calculate Fermi contact couplings of the rotor protons. All calculations were carried out in vacuum. For the geometry optimization we employed the B3LYP functional with the def2-TZVP basis set in combination with D3BJ dispersion correction. We used the same conditions to evaluate the rotation barriers of the methyl rotors on alkyl substituents with a relaxed (*V*^r^_3_) and a constrained (*V*^c^_3_) energy surface scan. In the relaxed scan, the molecule is allowed to avoid unfavourable interactions while changing the dihedral angle of the rotor whereas the constrained scan keeps all atom positions fixed in the geometry-optimized structure. Table 1 illustrates the results of the constrained and relaxed surface scan DFT-calculations. We obtained Fermi contact HF couplings *A*_FC_ of the rotor protons for the geometry-optimized structure using the eprnmr-module with the B3LYP functional, D3BJ dispersion correction and either the EPR-II or EPR-III basis set. The dipolar coupling constants *A*_DD_ were evaluated by determining *r*_*i*_ between the rotor proton position *i* and the approximated electron position in the middle of the N–O-bond from the geometry-optimized structure. The hyperfine couplings for the different rotors of the investigated nitroxides are shown in Table S1 in the ESI.† Additionally, the geometry-optimized structures of DENO and TEIO are illustrated in Fig. S1 (ESI†).

**Table 1:** DFT-calculated rotation barriers for the geometry-optimized molecular structures in vacuum. The rotation barriers from the relaxed (*V*^r^_3,*m*_) and constrained (*V*^c^_3,*m*_) energy surface scans are given for the relevant rotors *m* within the molecular structure. The average rotation barriers of the different surface scan methods are given as *V̄* for the nitroxides with only equivalent alkyl rotors. In case of the pyrroline-base nitroxides DENO and TEIO, all rotors *m* correspond to ethyl substituents. For the DOXYL nitroxides, *m* = 1, 2 correspond to methyl substituents and *m* = 3, 4 to ethyl, propyl, butyl or pentyl alkyl chains depending on the molecular structure. All values are given in Kelvin [K]

*m*	DENO		TEIO		cyc-DOXYL		2-DOXYL-C_11_		3-DOXYL-C_5_		3-DOXYL-C_14_		4-DOXYL-C_14_		5-DOXYL-C_14_		6-DOXYL-C_14_	
	*V* ^r^_3_	*V* ^c^_3_	*V* ^r^_3_	*V* ^c^_3_	*V* ^r^_3_	*V* ^c^_3_	*V* ^r^_3_	*V* ^c^_3_	*V* ^r^_3_	*V* ^c^_3_	*V* ^r^_3_	*V* ^c^_3_	*V* ^r^_3_	*V* ^c^_3_	*V* ^r^_3_	*V* ^c^_3_	*V* ^r^_3_	*V* ^c^_3_
1	1020	1504	1300	2279	1774	1972	1744	1954	1419	1601	1757	1940	1721	1959	1807	1969	1741	1966
2	1702	2454	1597	2224	1386	1551	1362	1511	1814	2032	1461	1590	1399	1575	1336	1508	1401	1548
3	1369	1550	1255	1487	—	—	1382	1631	1537	1560	948	1234	1464	1549	1423	1504	1424	1507
4	1332	1477	1322	1475	—	—	—	—	1263	1828	—	—	—	—	—	—	—	—
*V̄*	1356	1746	1369	1866	1580	1762	1496	1699	—	—	—	—	—	—	—	—	—	—

### 3.3 Inference of the rotation barrier distribution

Determining the underlying rotation barrier distribution from experimental Hahn echo decay data relies on numerical simulation of the tunneling ESEEM kernel. We adapted the density operator formalism implementations for the two-pulse ESEEM sequence^24^ in MATLAB^48^ to the investigated nitroxide structures and the experimental conditions. We assumed ideal pulses only in the kernel simulations. We simulated the kernel for a linear vector of rotation barriers *V*_3_, however in our simulation the tunneling Hamiltonian depends on the tunneling frequency *ν*_t_. Therefore, we calculated the corresponding (non-linear) vector of tunneling frequencies *ν*_t_ by diagonalizing the rotational Hamiltonian^32^ in a sufficiently large rotational basis (*r* = 0, …, 32). The Fermi contact and dipolar coupling constants evaluated from the geometry-optimized structure were included in the spin Hamiltonian. We ensured that the relative orientation of all methyl rotors *m* in the nitroxide structure are fixed during the simulation of the kernel. The orientation-dependence of the dipolar interaction was simulated with 841 orientations of the nitroxide with respect to the external magnetic field using a weighted grid generated by the sphgrid-function from EasySpin version 5.2.35.^49^ In Section S2 of the ESI,† we demonstrate the impact of considering the static Hamiltonian during the pulses as well as orientation selection on the tunneling ESEEM contribution and rationalize under which measurement conditions these effects must be considered.

Determining the rotation barrier distribution from the experimental two-pulse ESEEM data requires simplifications of the MQR model tailored to the investigated nitroxide. In case of only one or *M* chemically and magnetically equivalent methyl rotors,^24^ the expression of tunneling ESEEM contribution (see eqn (6)) simplifies to a Fredholm integral of the first kind9
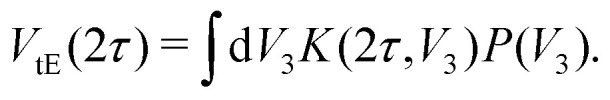
where *P*(*V*_3_) is a univariate distribution. We assume this for the rotors in DENO, TEIO, cyc-DOXYL and 2-DOXYL-C_11_. For paramagnetic molecules like the multi-substituent nitroxides 3-DOXYL-C_5_, 3-DOXYL-C_14_, 4-DOXYL-C_14_, 5-DOXYL-C_14_ and 6-DOXYL-C_14_ where different types of rotors are present, eqn (6) can only be simplified according to the number of equivalent rotor types. In case of the mixed-rotor DOXYLs (see Fig. 3), a minimum of two different rotor types are present in the structure and need to be considered in the tunneling ESEEM contribution according to10
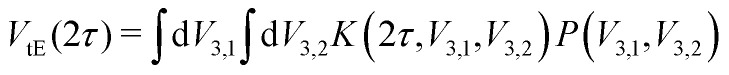
where *P*(*V*_3,1_,*V*_3,2_) represents a bivariate distribution. We attributed *V*_3,1_ to rotors on methyl substituents and *V*_3,2_ to the rotors in ethyl, propyl, butyl or pentyl groups in the different compounds. For the mixed-rotor nitroxides we additionally assumed no correlation between the rotation barrier distributions of the different rotor types. This further simplifies eqn (10), since the bivariate distribution *P*(*V*_3,1_, *V*_3,2_) can be expressed as the product of the univariate distributions *P*(*V*_3,1_)·*P*(*V*_3,2_) of the two rotor types.

We implemented a Gaussian model to characterize the rotation barrier distributions.^24^ The rotation barrier distribution can be evaluated by ordinary least-squares fitting11
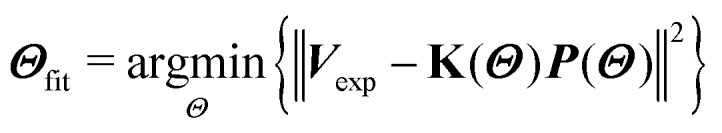
where ***Θ*** represents the vector of all MQR model parameters.^50^ The fitting procedure of the experimental Hahn echo decay data was performed using DeerLab version 1.0.1^50^ with Python 3.10.4.

## 4 Results and discussion

### 4.1 Nitroxides with methyl rotors in equivalent alkyl groups

#### 4.1.1 Influence of hyperfine approximations

**Fig. 4:**
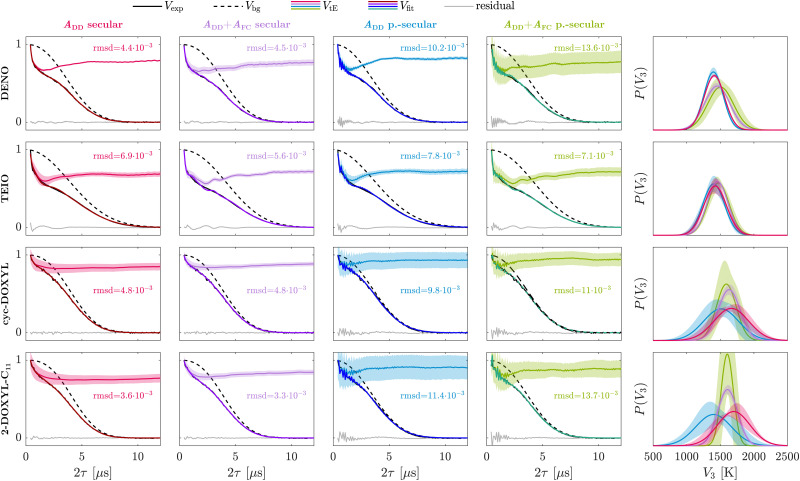
Extracted Gaussian rotation barrier distributions from experimental two-pulse ESEEM data of nitroxides with equivalent rotors for different hyperfine interaction approximations. The rotation barrier distributions were determined for experimental X-band data of DENO, TEIO, cyc-DOXYL and 2-DOXYL-C_11_ measured at *B*_max_ and a temperature of 20 K. Since these nitroxides contain equivalent alkyl groups, a univariate distribution was assumed. The tunneling ESEEM kernel implemented in the fitting procedure included the dipolar contribution only (*A*_DD_, red and blue) or both Fermi contact and dipolar contribution (*A*_FC_ + *A*_DD_, purple and green) in the secular hyperfine term *A*_*i*_(*ϕ*)*Ŝ*_*z*_*Î*_*i*,*z*_. The full state Hamiltonian used to simulate the tunneling ESEEM kernel contained only the secular hyperfine term for the first two columns (red and purple, secular), whereas for the third and forth column (blue and green, p.-secular) the Hamiltonian also contains the pseudo-secular hyperfine term *B*_*i*_(*ϕ*)*Ŝ*_*z*_*Î*_*i*,*x*_. The root-mean-square deviation (rmsd) serves as an additional metric to evaluate the time-domain fit quality. The last column represents an overlay of the extracted Gaussian rotation barrier distributions, where the colour indicates which hyperfine approximation was employed in the kernel. The shaded area represents the 95% covariance-based confidence interval.

The nitroxides DENO, TEIO, cyc-DOXYL and 2-DOXYL-C_11_ only contain equivalent methyl rotors. In case of DENO and TEIO the rotors are geminal ethyl groups whereas cyc-DOXYL and 2-DOXYL-C_11_ contain geminal methyl groups. We analyzed the experimental data assuming a univariate rotation barrier distribution in the MQR model using different hyperfine interaction approximations in the tunneling ESEEM kernel to understand their significance to the observed two-pulse tunneling ESEEM. Fig. 4 illustrates the fitting results for experimental data measured at X-band, at field position *B*_max_ and a temperature of 20 K for the mentioned nitroxides. For all nitroxides we observe differences in the time-domain fit quality for the examined HF approximations according to the root-mean-square deviation (rmsd).

For the methyl rotors in the ethyl groups of DENO, we find a decrease in goodness-of-fit of the Hahn echo decay signal when the isotropic Fermi contact hyperfine interaction is included in the tunneling ESEEM kernel in comparison to considering the dipolar interaction only (Fig. 4, red and purple). However, this difference is hardly significant. The rotation barrier distribution shows a shift to higher values and becomes broader upon inclusion of the Fermi contact term. It is important to note that the uncertainty of the tunneling ESEEM contribution as well as the inferred distribution increases when including the Fermi contact term. For TEIO, including the Fermi contact term leads to a smaller rmsd even though the fit residual contains more oscillations introduced by the fitted tunneling ESEEM contribution (Fig. 4). The inferred Gaussian rotation barrier distributions are almost identical for all hyperfine approximations. The uncertainty of the tunneling ESEEM and the rotation barrier distribution is higher when considering only the dipolar interaction for TEIO. This is contrary to the results for DENO, where including the Fermi contact term leads to more uncertainty of the tunneling contribution. In the following we try to identify the reason for these contradictory fitting results. DENO and TEIO have the same pyrroline-based nitroxide ring structure. Their only differing structural elements are the carboxyl group for DENO and the isoindoline ring extension for TEIO, which might impact the local hindrance of their ethyl groups differently. These slight intramolecular differences are visible in the constrained rotation barriers (see Table 1) and can be rationalized by looking at the geometry-optimized molecular structures of DENO and TEIO (Fig. S1 in the ESI†). However, the relaxed rotation barriers, where unfavourable intramolecular steric interactions are avoided, become similar for all rotors *m* in the nitroxide molecule (except for rotor *m* = 2), showcasing high conformational flexibility of the ethyl groups in DENO and TEIO. This conformational flexibility might suggest that calculating the Fermi contact interaction from the geometry-optimized structure for ethyl protons is not a good approximation, since the rigid molecular structure is not representative of the ensemble of nitroxide conformations present in the experimentally observed glass. Moreover, this might be the reason why the rmsd for DENO becomes worse when including the Fermi contact term but better for TEIO. Likewise, the point-dipole approximation for the anisotropic hyperfine interaction between the rotor protons and the electron spin for one fixed conformation is questionable since the ethyl groups have significant conformational flexibility. We argue that it is still a reasonable approximation that would be hard to improve upon, because modeling of the distribution of ethyl group rotamers in a glassy environment may not be reliable. We evaluated conformers for the ethyl groups of DENO and TEIO using their geometry-optimized structure and present the resulting distribution of dipolar coupling constants in Section S3 in the ESI.† The implemented dipolar coupling constants from the geometry-optimized structure represent characteristic values within the possible ethyl group conformations. For the Fermi contact interaction however, is not easy to estimate if the calculated coupling constants are representative for the conformational ensemble because that requires knowledge of the spin density, which is straightforward using DFT-methods but requires extensive computational time. While one could attempt to estimate the populations of rotamers by a molecular dynamics (MD) simulation in explicit solvent and to calculate full hyperfine couplings for all rotamers, we consider this effort as excessive. Such simulations would need to be performed at a temperature well above the glass transition, which would compromise the fidelity of the rotamer populations.

In case of the methyl rotors of cyc-DOXYL and 2-DOXYL-C_11_, we observe a slight improvement of the overall time-domain fit, based on the rmsd, when including the Fermi contact interaction in the Hamiltonian. The inferred rotation barrier distribution becomes narrower if the isotropic hyperfine interaction is considered and the confidence intervals of the tunneling ESEEM contribution are smaller for this approximation. In contrast to the ethyl groups, the methyl substituents are very rigid and have less conformational possibilities to avoid unfavourable steric interactions. This is also visible in the DFT-calculated rotation barriers for cyc-DOXYL and 2-DOXYL-C_11_ (see Table 1). There are significant differences in the constrained rotation barriers between the rotors *m* of cyc-DOXYL and 2-DOXYL-C_11_, respectively, which originate from the intramolecular local hindrance by the bulky residue. Unlike methyl rotors in ethyl groups, the rotation barriers evaluated using a relaxed surface scan still show different values for the rotors *m* within each nitroxide molecule, suggesting these groups cannot avoid unfavourable steric interaction. This in return leads to a larger and more significant isotropic HF interaction making the point-dipole approximation and including the Fermi contact terms for the geometry-optimized structure feasible. Therefore, we conclude that including the Fermi contact interaction for geminal methyl group rotors helps to represent the spin dynamics which are responsible for the observed tunneling ESEEM signal. However, for ethyl groups it is reasonable to approximate the secular hyperfine term with only the dipolar interaction.

Fig. 4 additionally shows the influence of the pseudo-secular hyperfine term on the Hahn echo decay fit (blue and green). Including the pseudo-secular term in the tunneling ESEEM kernel leads to additional nuclear ESEEM of the rotor protons in the tunneling ESEEM contribution. This results in high-frequency oscillations introduced by the fitting procedure at the beginning of the time-domain signal which are not observed experimentally. The goodness-of-fit decreases upon including the pseudo-secular term in the kernel and results in increased uncertainty of the tunneling ESEEM contribution as well as the inferred rotation barrier distribution. According to the rmsd and fit residuals, the best fits of the experimental Hahn echo decay signals are obtained considering only the secular HF term for the investigated nitroxides with equivalent alkyl groups.

#### 4.1.2 Differences in *P*(*V*_3_) for methyl rotors of geminal methyl and ethyl groups

We summarize the optimal fit parameters for DENO, TEIO, cyc-DOXYL and 2-DOXYL-C_11_ measured at *B*_max_ in the ESI,† Table S2. A difference in tunneling ESEEM can be observed for the different nitroxides in their experimental data (see Fig. 4). DENO and TEIO, which contain only methyl rotors in ethyl groups, show a larger tunneling modulation depth and slightly shorter modulation period than the methyl rotors of cyc-DOXYL and 2-DOXYL-C_11_. This observable difference in tunneling ESEEM is in agreement with the theory presented in Section 2.2, where we explained that the tunneling ESEEM matching condition differs for methyl rotors in different alkyl groups due to differences in their spin and tunneling Hamiltonian.

The inferred Gaussian rotation barrier distributions for DENO and TEIO measured at X-band and 20 K show mean values of *V*_av_ = 1411 K and *V*_av_ = 1423 K, respectively. For both nitroxides, these mean barrier values lie close to the average of the DFT-calculated relaxed scan rotation barriers *V̄*^r^_3_(DENO) = 1356 K and *V̄*^r^_3_(TEIO) = 1369 K (see Table 1). The distribution widths for DENO and TEIO were evaluated at *σ* = 152 K and *σ* = 168 K, respectively. The rotation barrier distributions of these ethyl-containing nitroxides are very similar. The inferred rotation barrier distributions indicate that the ensemble of nitroxide conformations is comparable for DENO and TEIO and also the glass-dependent local environments in both samples are similar. The nitroxide ring structure for both systems is pyrroline-based and the relaxed rotation barriers are comparable for DENO and TEIO (see Table 1), indicating that they experience similar intramolecular local hindrance in vacuum when unfavourable steric interactions can be avoided. Since TEIO and DENO are also investigated in the same matrix, similar glass-dependent local environments can be expected, which would overall likely result in very similar rotation barrier distributions.

The geminal methyl group containing nitroxides cyc-DOXYL and 2-DOXYL-C_11_ exhibit a less pronounced tunneling ESEEM than the ethyl group containing DENO and TEIO (see Fig. 4). The inferred Gaussian rotation barrier distributions for cyc-DOXYL and 2-DOXYL-C_11_ show mean values of *V*_av_ = 1630 K and *V*_av_ = 1607 K, respectively. Their distribution widths were evaluated at *σ* = 163 K for cyc-DOXYL and *σ* = 147 K for 2-DOXYL-C_11_. Generally, the mean rotation barriers for both methyl group containing nitroxides are located between the DFT-calculated relaxed and constrained rotation barrier averages (see Table 1). Additionally, the inferred rotation barrier distributions for cyc-DOXYL and 2-DOXYL-C_11_ are very similar. The DFT-calculated relaxed and constrained rotation barriers are also almost identical for cyc-DOXYL and 2-DOXYL-C_11_, indicating that the methyl rotors *m* of both nitroxides experience very similar intramolecular hindrance (see Table 1). Since the EPR experiments for these methyl rotor containing nitroxides were carried out in the same glassy matrix, the glass environment exhibits also very similar local hindrance on the methyl rotors of both nitroxides, rationalizing the similarity between the inferred rotation barrier distributions of cyc-DOXYL and 2-DOXYL-C_11_. The overall tunneling ESEEM modulation depth *Δ* of 2-DOXYL-C_11_ (*Δ* = 21.2%) is around 5% larger than for cyc-DOXYL (*Δ* = 15.7%) suggesting that the tunneling ESEEM modulation depth of *M* methyl rotors approximately follows the product rule *Δ* = 1 − (1 − *k*)^*M*^ adapted from dipolar coupling studies between electron spins,^51^ where *k* represents the modulation depth from methyl group *m*. This explains the larger modulation depth for 2-DOXYL-C_11_, because it contains three methyl rotors compared to cyc-DOXYL which only has two methyl rotors.

As the DFT-calculations suggest, the rotation barriers for geminal methyl group rotors in nitroxides are higher than for methyl rotors in ethyl groups, which our extracted Gaussian rotation barrier distributions for cyc-DOXYL and 2-DOXYL-C_11_ confirm. This suggests that methyl rotors in ethyl groups experience less hindrance than the rotors of geminal methyl group, which we rationalize by the additional conformational flexibility of ethyl groups. More rotational degrees of freedom allows the methyl rotor in the ethyl group to avoid unfavourable steric interactions manifesting in a lower rotation barrier distribution. For classical rotation, the barrier is also found to be higher for geminal methyl group rotors than for the methyl rotors of ethyl groups attached to a nitroxide ring (see ESI,† Section S5). To ensure that using a Gaussian model does not impose bias on the rotation barrier distribution, we fitted the experimental data of DENO, TEIO, cyc-DOXYL and 2-DOXYL-C_11_ using a non-parametric model for *P*(*V*_3_). The results are illustrated in Fig. S6 in the ESI† and confirm our interpretations based on the Gaussian distributions. Our analysis reveals that the MQR model is a sensitive method to quantify differences in ESEEM-detected tunneling behaviour using the inferred rotation barrier distribution.

Here, we want to point out that the extracted rotation barriers for cyc-DOXYL and 2-DOXYL-C_11_ are lower than for the study performed on H-mNOHex in *ortho*-terphenyl (*V*_3,av_ ∼ 1730 K at 20 K).^24^ The nitroxides investigated here are based on a proxyl ring structure, whereas H-mNOHex is based on pyrroline. Moreover, the glassy matrix is not the same in these studies and might also influence the observed tunneling ESEEM. Additionally, the Fermi contact interaction was not included in the tunneling ESEEM kernel for the analysis of H-mNOHex, since we only became aware of its relevance in this study. We re-analyzed all data presented in our previous study^24^ and found that the interpretations and conclusions are still valid. Therefore, the results of this and our previous work^24^ are not contradictory, but a systematic study on the influence of the nitroxide ring structure and the glassy matrix on the tunneling ESEEM and rotation barrier distribution might provide revealing insights.

### 4.2 Nitroxides with methyl rotors in inequivalent alkyl groups

#### 4.2.1 Identifiability analysis of bivariate MQR model

Mixed-rotor nitroxides contain chemically and magnetically inequivalent methyl rotors on different alkyl groups as in 3-DOXYL-C_5_, 3-DOXYL-C_14_, 4-DOXYL-C_14_, 5-DOXYL-C_14_ and 6-DOXYL-C_14_ (see Fig. 3). Investigating the tunneling ESEEM originating from different rotor types requires including a bivariate distribution in the MQR model which accounts for the rotation barriers expected for the different alkyl rotors. This increases the overall parameter space evaluated during the least-squares fitting. It is of great importance that all model parameters ***Θ*** are identifiable,^52^ which means that only a single global minimum of the objective function *F*(***Θ***) exists.

12 *F*(***Θ***) = ‖***V***_exp_ − ***V***_fit_(***Θ***)‖^2^

Assessing the likelihood profiles^53^ of all parameters in the model according to13
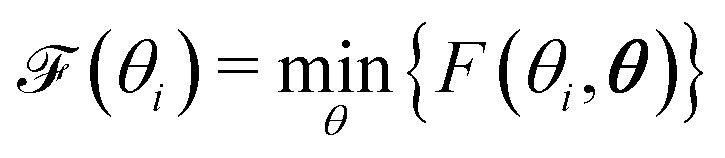
provides a graphical and quantitative representation of the identifiability of an individual parameter by minimizing the objective function for all parameters *θ*_*j*≠*i*_ for fixed values of the parameter of interest *θ*_*i*_.^54^ The likelihood profile for an identifiable parameter shows a single well-defined minimum, whereas a non-identifiable parameter might exhibit several equivalent global minima or a flat region corresponding to the minimum of the objective function. The likelihood profile also gives insight on the significance of the parameter in the employed model.

**Fig. 5:**
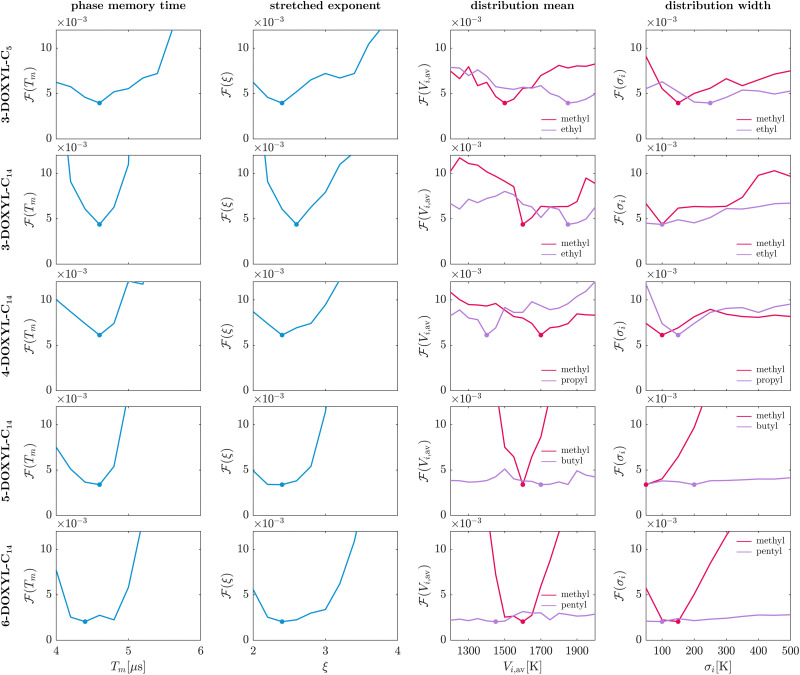
Likelihood profiles of all model parameters in the bivariate MQR model employed for mixed-rotor nitroxides. The phase memory time *T*_m_ and the stretch parameter *ξ* characterize the matrix-driven decoherence contribution (blue). The tunneling ESEEM contribution relies on the mean value *V*_*i*,av_ and standard deviation *σ*_*i*_ to parameterize the rotation barrier distribution of rotor type *i* (*i* = 1 – red, *i* = 2 – purple). Their likelihood profiles indicate the impact of these parameters on the overall goodness-of-fit. The dots mark the minimum of the likelihood profiles.

Fig. 5 presents the likelihood profiles for all fit parameters employed in the bivariate MQR model for the mixed-rotor DOXYL compounds. The likelihood profiles of the phase memory time *T*_m_ and the stretch parameter *ξ* characterizing the matrix-driven decoherence contribution show distinct global minima for all nitroxides, meaning these parameters are identifiable. This is not surprising, since the two-pulse ESEEM sequence excites electron spin coherence that undergoes relaxation leading to a decaying signal. In the employed model, we use the stretched exponential background function *V*_bg_ to account for the decay induced by nuclear pair ESEEM of matrix protons.^19,26^

The likelihood profiles for the tunneling ESEEM parameters *V*_*i*,av_ and *σ*_*i*_ for the investigated mixed-rotor nitroxides are also illustrated in Fig. 5. Since both the distribution mean *V*_*i*,av_ and the distribution width *σ*_*i*_ describe the Gaussian rotation barrier distribution of rotor type *i*, we will discuss the identifiability of the inferred distributions *P*(*V*_3,*i*_) rather than for the individual parameters. All mixed-rotor nitroxides contain two geminal methyl group rotors, which are attributed to rotor type *i* = 1 (red, Fig. 5). The distribution parameters of rotor type *i* = 2 correspond to methyl rotors in ethyl groups for 3-DOXYL-C_5_ and 3-DOXYL-C_14_, in propyl groups for 4-DOXYL-C_14_, in butyl groups for 5-DOXYL-C_14_ and in pentyl groups for 6-DOXYL-C_14_ (purple, Fig. 5).

For 3-DOXYL-C_5_, 3-DOXYL-C_14_ and 4-DOXYL-C_14_ the distribution parameters of the geminal methyl group rotors show a global minimum, however the likelihood profiles are rather flat compared to the ones for the phase memory time and stretch parameter. Similar to the geminal methyl group rotor parameters of these three DOXYL compounds, the distribution parameters of their methyl rotors in ethyl and propyl groups show a distinct minimum in the identifiability analysis, but also their likelihood profiles are quite flat. For 3-DOXYL-C_5_, which contains two geminal methyl group rotors and two methyl rotors in ethyl groups, the identifiability analysis suggests that both rotor types contribute to the observed tunneling ESEEM since the objective function changes when varying the distribution parameters of both rotors. A similar interpretation can be drawn from the likelihood profiles of 3-DOXYL-C_14_, which comprises also two geminal methyl group rotors but only one methyl rotor in an ethyl group. However, for both nitroxides their *V*_2,av_-profiles imply that the optimal goodness-of-fit using a bivariate distribution model is found for a methyl rotor distribution mean *V*_2,av_ ∼ 1850 K in ethyl groups. This does not agree with the DFT-calculations for 3-DOXYL-C_5_ and 3-DOXYL-C_14_ that predicted rotation barriers for their methyl rotors in ethyl groups in the range of 1000–1550 K with the relaxed surface scan (see Table 1). We will further discuss these contradictory results in Section 4.2.2. The identifiability analysis of 4-DOXYL-14 clearly shows that both the geminal methyl group rotors and methyl rotors in propyl groups have a significant impact on the objective function, and their profile minima agree with the DFT-calculated rotation barriers in Table 1. In this case, both rotor types must be considered when analysing the experimental tunneling ESEEM. For 5-DOXYL-C_14_ and 6-DOXYL-C_14_ the likelihood profiles of their geminal methyl rotor distribution parameters show distinct and well-defined minima indicating that the geminal methyl group rotors are relevant for fitting the tunneling ESEEM contribution of these two nitroxides. The methyl rotors in butyl and pentyl groups of 5-DOXYL-C_14_ and 6-DOXYL-C_14_, respectively, are irrelevant for fitting the tunneling ESEEM contribution since their evaluated likelihood profiles are practically constant. This indicates that the optimal time-domain fit with the bivariate MQR model can be found for any value of *V*_2,av_ and *σ*_2_. Therefore, it is unnecessary to use a bivariate distribution in the MQR model when inferring their rotation barrier distributions from experimental data.

The identifiability analysis of all parameters employed in the bivariate MQR model suggest that geminal methyl group rotors as well as methyl rotors in ethyl and propyl groups contribute to the observed tunneling ESEEM in mixed-rotor nitroxides, since they all show rotation barrier distribution likelihood profiles with a single global minimum for their parameters *V*_*i*,av_ and *σ*_*i*_. Tunneling from methyl rotors in butyl and pentyl groups has no impact on the overall goodness-of-fit using the bivariate MQR model revealing that these rotors do not exhibit substantial tunneling ESEEM. Overall, the results of the identifiability analysis are consistent with the theory on the tunneling ESEEM matching condition in Section 2.2 where we predicted which alkyl groups show significant tunneling ESEEM for the investigated nitroxide systems.

#### 4.2.2 Bivariate *P*(*V*_3_) using Monte-Carlo sampling

Since theoretically for the investigated mixed-rotor nitroxides a bivariate distribution should be employed in the MQR model, the parameter space evaluated by the least-squares fitting algorithm to find the underlying rotation barrier distribution is enormous. This results in unreliable fitting solutions that strongly depend on the starting values of the distribution parameters *V*_1,av_, *V*_2,av_, *σ*_1_ and *σ*_2_. Therefore, we alternatively rely on Monte-Carlo (MC) sampling of the parameter space while monitoring the objective function (eqn (12)) to find optimal parameters for the experimental data with the bivariate MQR model.

**Fig. 6:**
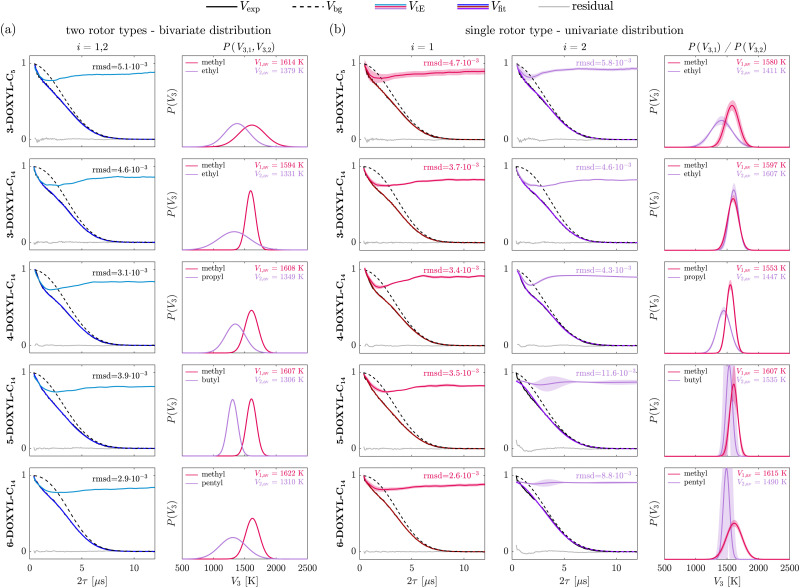
Inferred Gaussian rotation barrier distributions for nitroxides with methyl rotors in inequivalent alkyl groups using different MQR models. Experimental two-pulse ESEEM data recorded at *B*_max_ and 20 K of 3-DOXYL-C_5_, 3-DOXYL-C_14_, 4-DOXYL-C_14_, 5-DOXYL-C_14_ and 6-DOXYL-C_14_ (solid black lines) were fitted using two different MQR models. (a) Both rotor types *i* = 1, 2 were considered in the MQR model, which required implementation of a bivariate rotation barrier distribution. (b) Only a single rotor type (either *i* = 1 or *i* = 2) was considered in the MQR model, which therefore required a univariate distribution. In (a) the time-domain fits were evaluated by constrained Monte-Carlo (MC) sampling of the parameter space using 50’000 samples whereas in (b) a least-squares fitting algorithm was employed to find the optimal fit parameters. The root-mean-square deviation (rmsd) serves as an additional metric to evaluate the time-domain fit quality. The shaded areas represent the 95% covariance-based confidence intervals.

Fig. 6(a) shows the fits using 50’000 Monte-Carlo samples evaluated for 3-DOXYL-C_5_, 3-DOXYL-C_14_, 4-DOXYL-C_14_, 5-DOXYL-C_14_ and 6-DOXYL-C_14_ measured at *B*_max_ and 20 K. We implemented boundary conditions on the distribution means of *V*_1,av_ = [1500 K, 1800 K] and *V*_2,av_ = [1200 K, 1500 K] according to their DFT-calculated rotation barriers as well as our findings from the analysis of cyc-DOXYL, 2-DOXYL-C_11_, DENO and TEIO. We will refer to this MC sampling fitting procedure as constrained, because it ensures that the rotation barrier distribution for geminal methyl group rotors lies at higher values than the distribution of methyl rotors in ethyl, propyl, butyl and pentyl groups. The results for all mixed-rotor DOXYLs show mean values *V*_1,av_ in the range of 1590–1620 K for the rotation barrier distribution of the geminal methyl group rotors with varying distribution widths *σ*_1_ between 75 K and 115 K. Only 3-DOXYL-C_5_ shows a broader rotation barrier distribution (*σ*_1_ = 209 K). For all mixed-rotor nitroxides, *V*_1,av_ is in very good agreement with the extracted rotation barrier distribution for cyc-DOXYL (*V*_av_ = 1630 K and *σ* = 163 K). This is expected, since they all have the same nitroxide ring structure and the geminal methyl group rotors are attached in the same position. The extracted rotation barrier distribution for the two methyl rotors in ethyl groups in 3-DOXYL-C_5_ shows a mean of 1379 K and a similar width as its geminal methyl group rotor distribution. Similar distribution means of 1309 K and 1349 K are found for the methyl rotor in the ethyl group of 3-DOXYL-C_14_ and the methyl rotor in the propyl group of 4-DOXYL-C_14_, respectively. Their distribution width is comparable to the methyl rotor distribution in ethyl groups of 3-DOXYL-C_5_, but *σ*_2_ is larger than the width of their respective geminal methyl rotor distribution for both 3-DOXYL-C_14_ and 4-DOXYL-C_14_. For 5-DOXYL-C_14_ and 6-DOXYL-C_14_, the rotation barrier distribution in their butyl and pentyl groups also show lower *V*_2,av_ of 1306 K and 1310 K, respectively, than their methyl rotor distributions in geminal methyl groups. Generally, comparing the rotation barrier distributions for geminal methyl group rotors and methyl rotors in ethyl groups of the mixed-rotor DOXYLs with the results for cyc-DOXYL, 2-DOXYL-C_11_, DENO and TEIO confirms that the constrained MC sampling fitting procedure extracts reasonable bivariate distributions from the experimental data.

The results obtained from the constrained MC sampling fitting reveal different rotation barrier distributions for the methyl rotors in ethyl groups in 3-DOXYL-C_5_ and 3-DOXYL-C_14_ than our identifiability analysis predicted in Section 4.2.1. The likelihood profiles of the methyl rotors in ethyl groups suggest a distribution mean of *V*_2,av_ ∼ 1850 K. Monte-Carlo sampling relies on setting boundaries to all model parameters, which might bias the fitting procedure and influence which solution is considered optimal for the experimental data. Therefore, it is not surprising that the constrained MC fitting procedure is unable to find a solution with such a high rotation barrier for the methyl rotors in ethyl groups, since we only allowed mean values in the range between 1200–1500 K. Therefore, we compared the constrained MC results with an alternative MC sampling fit, that employs parameter boundaries for the mean values of the bivariate distribution of *V*_*i*,av_ = [1200 K, 2000 K]. This covers the full range of distribution means investigated in the identifiability analysis. The results of this unconstrained MC fitting procedure are listed in Table S3 in the ESI.† The optimal parameter values evaluated with the unconstrained MC fitting agree with the minima of the likelihood profiles. For all DOXYL compounds, the fit quality is equally as good or even better for the unconstrained than for the constrained MC fitting according to the rmsd. These results suggest that independent of the *V*_*i*,av_ boundary conditions (constrained or unconstrained) for the MC fitting, solutions with overall good fit quality can be found using a bivariate distribution model. This might indicate that the distribution parameters can compensate each other to minimize the objective function and increase the fit quality regardless of whether the solution truly captures the underlying spin dynamics. If this is the case, the same interpretation is valid for the evaluated rotation barrier distribution likelihood profiles. This would explain why both the ethyl distribution likelihood profiles of 3-DOXYL-C_5_ and 3-DOXYL-C_14_ as well as their unconstrained MC fit suggest an optimal Hahn echo decay fit for very high rotation barriers for the methyl rotors in ethyl groups, which do not agree with the DFT-calculations. This makes the interpretation of the extracted rotation barrier distributions by the MC sampling procedure unreliable especially for 3-DOXYL-C_5_, 3-DOXYL-C_14_ and 4-DOXYL-C_14_.

Therefore, we additionally fitted the experimental Hahn echo decay data considering a single rotor type *i* (*e.g.* only the rotors of the methyl groups) in the MQR model using a univariate rotation barrier distribution. If it suffices to fit the experimental signal when considering only one rotor type, this indicates that the tunneling ESEEM is dominated by the rotation barrier distribution of this rotor type. Rotor type *i* = 1 always corresponds to the geminal methyl group rotors, while *i* = 2 represents the methyl rotors in ethyl (3-DOXYL-C_5_, 3-DOXYL-C_14_), propyl (4-DOXYL-C_14_), butyl (5-DOXYL-C_14_) and pentyl groups (6-DOXYL-C_14_). Fig. 6(b) shows the time-domain fit results and extracted univariate rotation barrier distributions when considering only rotor type *i* in the MQR model. For 3-DOXYL-C_5_, the time-domain fit quality for the single rotor type MQR model considering either methyl or ethyl groups is very similar to the constrained bivariate MC fit. Moreover, the extracted univariate rotation barrier distributions show very similar mean values to the bivariate distribution, but are narrower in width (see Fig. 6). Additionally, the goodness-of-fit between the MQR models considering the two rotor types separately is comparable, which makes it impossible to determine the dominant rotor type in the observed tunneling ESEEM contribution. The same interpretations are valid when analyzing the univariate fit results for 3-DOXYL-C_14_ and 4-DOXYL-C_14_, where the goodness-of-fit is very similar when considering the rotor types separately. For 3-DOXYL-C_14_, which contains two geminal methyl group rotors but only one methyl rotor in an ethyl group, we observe that the extracted univariate rotation barrier distribution for the methyl rotor in the ethyl group is almost identical to the geminal methyl group distribution. This is different from the bivariate distribution extracted with the constrained MC procedure, where the ethyl distribution shows lower rotation barriers than the methyl distribution. We suspect that the MQR model only considering the ethyl group leads the least-squares fitting algorithm to evaluate a rotation barrier distribution at unreasonably high values. This indicates that the methyl rotor of the single ethyl group in 3-DOXYL-C_14_ can not be solely responsible for the observed tunneling ESEEM. 4-DOXYL-C_14_ shows excellent rmsd for both rotor types (in methyl and propyl groups) using a univariate distribution model. The univariate rotation barrier distributions are comparable to the bivariate distribution extracted with the constrained MC procedure. Since the fit quality for the mixed-rotor nitroxides 3-DOXYL-C_5_, 3-DOXYL-C_14_ and 4-DOXYL-C_14_ is very good using either the univariate or the bivariate model, our analysis procedure does not allow us to identify if only the geminal methyl groups, only the ethyl or propyl groups or both rotor types simultaneously are responsible for the observed tunneling ESEEM. Therefore, it is unreasonable to extract quantitative local environment information from the experimental tunneling ESEEM of these nitroxides, because the methyl rotors of methyl, ethyl and propyl groups all can exhibit significant tunneling ESEEM and we can not infer which distribution model represents the underlying tunneling behaviour.

The extracted univariate rotation barrier distributions for the rotors of 5-DOXYL-C_14_ and 6-DOXYL-C_14_ reinforce our interpretations of their constrained MC fitting results as well as distribution likelihood profiles. The univariate model considering only the methyl group rotors shows excellent goodness-of-fit according to the evaluated rmsd, which is even better than the best fit of the constrained MC procedure shown in Fig. 6(a). Moreover, the univariate fits considering the methyl rotors of the butyl group in 5-DOXYL-C_14_ and pentyl group in 6-DOXYL-C_14_ reveal that these rotors do not contribute to the observed tunneling ESEEM. This can be observed in the fitted tunneling ESEEM contribution, which fails to fit the fast decaying tunneling contribution in the beginning of the signal, in the high rmsd value and in the uncertainty of the extracted rotation barrier distribution. Even though for these mixed-rotor nitroxides a bivariate distribution model is not necessary, the extracted methyl rotor rotation barrier distributions are consistent, which supports the presented theory and our implemented MC fitting approach.

The different fitting procedures provided insight in the underlying spin dynamics responsible for the experimentally observed tunneling ESEEM. It confirmed that rotors in methyl, ethyl and propyl groups exhibit significant tunneling ESEEM like we qualitatively predicted by analyzing the tunneling ESEEM matching condition for the methyl rotors of different alkyl groups. Furthermore, this combined approach allowed identification of rotor types that dominate the observed tunneling ESEEM contribution in mixed-rotor systems. In combination with the identifiability analysis, the introduced fitting approaches are crucial to understand the tunneling behaviour observed in Hahn echo decay signals originating from different rotors coupled to the observed electron spin.

## 5 Conclusion

In this work, we have demonstrated that methyl rotors in different alkyl groups in nitroxides exhibit characteristic tunneling ESEEM modulation depth and periods. These depend on the matching between the differences in rotor proton hyperfine coupling constants and the tunneling frequency of the rotor. We explored different hyperfine approximations in the spin Hamiltonian to understand their impact on the tunneling ESEEM contribution of geminal methyl group rotors and methyl rotors in ethyl groups. Our systematic analysis of the implemented hyperfine approximations revealed that the Fermi contact interaction must be considered for geminal methyl group rotors in nitroxides, while the dipolar interaction suffices to represent the tunneling ESEEM of methyl rotors in ethyl and longer alkyl groups of nitroxides.

We applied the MQR model to extract the underlying rotation barrier distribution from the experimental Hahn echo decay signals of nitroxides containing only methyl groups or ethyl groups. Thereby, we found that methyl rotors in ethyl groups experience less local hindrance manifesting in a lower rotation barrier distribution than for geminal methyl group rotors in nitroxides. This indicates that methyl rotors in ethyl groups can avoid unfavourable steric interactions by more rotational degrees of freedom which reflects in a lower mean rotation barrier. Thus, we have shown that the MQR model is able to quantify differences in rotor environment by the extracted rotation barrier distribution.

Additionally, we have extended the MQR model to treat tunneling ESEEM arising from different rotor types coupled to the same electron spin. Therefore, we included a bivariate rotation barrier distribution model and used a combination of different fitting approaches to determine the dominant rotor types responsible for the experimentally observed tunneling ESEEM signal. The identifiability analysis of all model parameters in the extended MQR model for mixed-rotor systems served as an important tool to understand the solutions of the different fitting approaches. Through a systematic study of mixed-rotor DOXYLs, we found that if geminal methyl group rotors are coupled to the same electron spin as methyl rotors in ethyl and propyl groups, both rotor types exhibit significant tunneling ESEEM. Monte-Carlo sampling of a constrained model parameter space enabled the inference of bivariate distributions for these mixed-rotor DOXYL compounds which agree with the distributions extracted for nitroxides containing only one of the rotor types. Moreover, we confirmed that methyl rotors in butyl and pentyl groups do not fulfill the required matching condition and therefore do not exhibit detectable tunneling ESEEM.

In this work, we have demonstrated that quantum rotor ESEEM spectroscopy is a sensitive and quantitative technique to investigate the local environment of methyl rotors in the low-temperature regime. Our study showcases the applicability of the MQR model to gain insight into tunneling behaviour of quantum rotors beyond methyl groups in nitroxides. In the future, tunneling ESEEM is a promising coherent phenomenon that has the potential to unveil structural information with help of the omnipresent methyl groups in amino acids of proteins. Quantification of their local environment with the MQR model could offer exciting insights into the short range structural conformation of spin-labelled proteins.

## Author contributions

Conceptualization: AE, GJ, SSE, GRE. Methodology: AE. Experiments and investigation: TN, SSE, GRE. Data analysis: AE. Visualization: AE. Validation: AE, GJ, SSE, GRE. Supervision: GJ, SSE, GRE. Funding acquisition: GJ, SSE, GRE. Writing – original draft: AE. Writing – review & editing: all authors.

## Conflicts of interest

There are no conflicts to declare.

## Supplementary Material

CP-026-D4CP01212G-s001

CP-026-D4CP01212G-s002
