# Supplementary material for: Exploring tunneling ESEEM beyond methyl groups in nitroxides at low temperatures
Source: Phys Chem Chem Phys. 2024 May 7;26(21):15240–54. doi: 10.1039/d4cp01212g (PMC11135458; doi:10.1039/d4cp01212g)
Supplement: CP-026-D4CP01212G-s002 [file CP-026-D4CP01212G-s002.pdf]

Electronic Supplementary Material (ESI) for Physical Chemistry Chemical Physics.
This journal is © the Owner Societies 2024

Supporting Information:

Exploring tunneling ESEEM beyond methyl groups in nitroxides at low temperatures

Andrea Eggeling,[a] Thacien Ngendahimana,[b] Gunnar Jeschke,[a*] Gareth R. Eaton,[b] and Sandra S. Eaton[b*]

[a] *ETH Zurich, Department of Chemistry and Applied Biosciences, Vladimir-Prelog-Weg 2, 8093 Zurich, Switzerland.*
[b] *University of Denver, Department of Chemistry and Biochemistry, Denver, CO 80208, USA.*

Contents

S1 DFT-calculations of the investigated nitroxides 2

S2 Influence of real pulses and selective excitation on the tunneling ESEEM contribution 3

S3 Ethyl group conformers of DENO and TEIO 5

S4 Fitted model parameters for equivalent-rotor nitroxides 5

S5 Impact of classical rotation on T_m for methyl and ethyl-containing nitroxides 6

S6 Comparison of Gaussian and non-parametric $P(V_3)$ for equivalent-rotor nitroxides 6

S7 Fitted model parameters for mixed-rotor nitroxides using different distribution models 8

S1 DFT-calculations of the investigated nitroxides

We used density functional theory (DFT) calculations to assess contributions to the hyperfine interaction between the rotor protons and the electron spin of the nitroxides. The isotropic Fermi contact interaction A_{FC} was evaluated with the `eprnmr`-module in ORCA using the B3LYP density functional with either the EPR-II or EPR-III basis set and in combination with the D3BJ dispersion correction. The results in Tab. 1 show that for all geminal methyl group rotors there is significant coupling strength for all three protons of the rotor. However, for the methyl rotors in ethyl groups (e.g. DENO, $m = 1-4$ and TEIO, $m = 1-4$) only one or two of the rotor protons show a significant Fermi contact interaction. For the methyl rotor in the propyl group of 4-DOXYL-C_{14} ($m = 3$), the isotropic hyperfine interaction of all protons becomes smaller than 1 MHz, and for the methyl rotors in butyl and pentyl groups in 5-DOXYL-C_{14} and 6-DOXYL-C_{14}, respectively, the interaction becomes negligible.

The distance-dependent dipolar hyperfine interaction A_{DD} was calculated by the point-dipole approximation from the geometry-optimized structure of the investigated nitroxides. The electron spin was approximated in the middle of the N-O-bond. The resulting dipolar hyperfine constants are listed in Tab. 1. Similar to the Fermi contact constants, the dipolar coupling constants are largest for the methyl rotor protons, since they are close in distance to the electron spin (DOXYLs, $m = 1,2$). For the methyl rotors in ethyl groups, the dipolar interaction strongly depends on the geometry-optimized structure, specifically in which direction the ethyl groups are pointing with respect to the N-O-bond. This is clearly visible from the geometry-optimized structures of DENO and TEIO, which are illustrated in Fig. S1. For DENO, the ethyl group $m = 4$ points down and away from the electron spin resulting in dipolar coupling constants below 1 MHz. Similar for TEIO, the ethyl groups $m = 1,2$ point down to the organic ring structure which manifests in a dipolar coupling around 1 MHz. For rotors of propyl groups and longer alkyl chains, the dipolar interaction becomes even smaller because the interspin distance between the coupling partner increases. The xyz-files of the geometry-optimized structures of all nitroxides are provided as a zip-file.

Table 1 DFT-calculations of hyperfine coupling constants for the geometry-optimized molecular structures in vacuum. The isotropic Fermi contact contribution (A_{FC}) and the dipolar contribution (A_{DD}) to the methyl proton-electron hyperfine couplings were calculated for the relevant methyl rotors m within the molecular structure. In case of the pyrroline-base nitroxides DENO and TEIO, all m correspond to ethyl substituents. For the DOXYL nitroxides, $m = 1,2$ correspond to methyl substituents and $m = 3,4$ to ethyl, propyl, butyl or pentyl alkyl chains depending on the molecular structure.

nitroxide		DENO				TEIO				cyc-DOXYL		2-DOXYL-C_{11}			3-DOXYLC$_5$			
m		1	2	3	4	1	2	3	4	1	2	1	2	3	1	2	3	4
A_{FC} [MHz]	H1	2.16	0.10	-0.04	-0.03	0.50	-0.03	1.77	-0.02	-1.71	-1.34	-1.58	-1.39	-1.13	-1.57	-1.55	0.91	-0.24
	H2	0.21	2.33	2.85	2.97	2.72	2.99	3.68	-0.09	-2.87	0.09	5.73	-1.67	4.06	1.11	2.56	3.24	0.03
	H3	2.24	2.13	1.46	1.01	-0.14	0.76	-0.05	0.03	5.36	-1.65	-2.79	0.23	-2.02	-1.90	4.30	-0.10	0.06
A_{DD} [MHz]	H1	3.14	1.59	1.80	0.93	0.80	0.82	4.68	3.80	2.82	3.25	2.75	3.37	2.97	3.36	2.75	0.84	1.18
	H2	4.55	1.21	1.19	0.74	0.74	0.76	1.21	1.16	4.25	1.62	1.56	4.27	1.57	1.64	4.08	0.69	4.02
	H3	1.26	4.11	4.53	0.84	0.95	0.91	1.89	1.58	1.59	4.24	4.06	1.63	4.04	4.32	1.56	0.89	1.70

nitroxide		3-DOXYL-C_{14}			4-DOXYL-C_{14}			5-DOXYL-C_{14}			6-DOXYL-C_{14}		
m		1	2	3	1	2	3	1	2	3	1	2	3
A_{FC} [MHz]	H1	-1.80	-1.37	0.03	-1.89	-1.37	0.79	-1.63	-1.33	-0.04	-1.78	-1.36	0.01
	H2	3.93	-1.69	1.11	-2.93	-0.32	0.48	-2.63	1.08	0.34	5.79	-1.64	0.00
	H3	-2.73	0.54	1.62	5.66	-1.60	0.00	6.85	-1.60	0.01	-2.90	-0.23	-0.00
A_{DD} [MHz]	H1	2.97	3.16	4.51	2.74	3.34	0.56	2.70	3.43	0.25	2.75	3.37	0.25
	H2	1.62	4.18	1.21	4.18	1.64	0.56	3.97	1.64	0.20	1.57	4.29	0.18
	H3	4.41	1.61	2.71	1.58	4.30	0.84	1.54	4.32	0.24	4.14	1.64	0.20

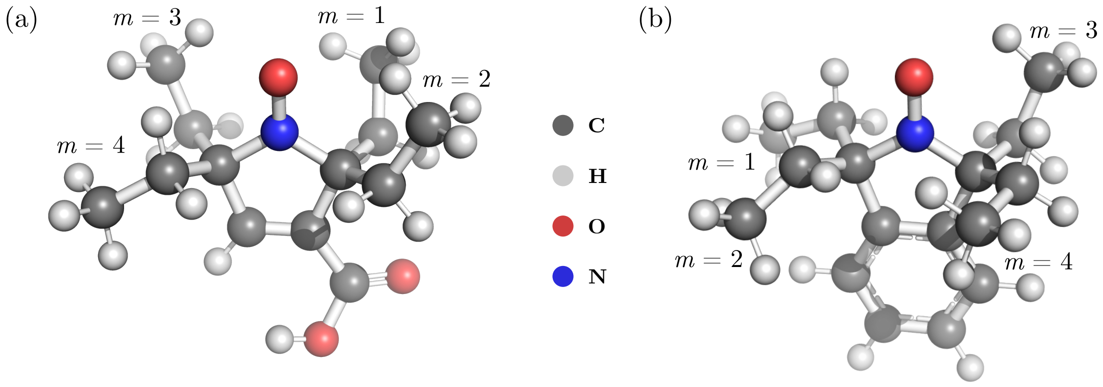

Fig. S1 Geometry-optimized molecular structures of (a) DENO and (b) TEIO. The methyl rotors m in ethyl groups are marked with their numbers used for the DFT-calculations of the relaxed and constrained energy surface scans to evaluate their rotation barriers, and for the calculations of their rotor's proton hyperfine interactions. The structures are visualized using PyMOL[1].

S2 Influence of real pulses and selective excitation on the tunneling ESEEM contribution

In a glassy matrix, the nitroxide electron spin exhibits an anisotropic spectrum dominated by the g-tensor and the hyperfine coupling tensor to the ^{14}N-nucleus. The spectrum at both X- and Q-band frequency is typically broader than the maximal excitation bandwidth accessible with pulses. This implies that only a certain region of the EPR-spectrum is excited along with off-resonance excitation that occurs due to the anisotropy of the spin system. Since the resonance offset Ω as well as the hyperfine interaction between the electron spin and the methyl proton are orientation-dependent, we want to investigate the influence of selective excitation of the electron spin spectrum on the tunneling ESEEM contribution. Therefore, we need to include the resonance offset in the spin Hamiltonian. The resonance offset is mainly dictated by the effective g- and A(^{14}N)-values

$$g_{\text{eff}} = \sqrt{g_x^2 \sin^2\theta \cos^2\varphi + g_y^2 \sin^2\theta \sin^2\varphi + g_z^2 \cos^2\theta} \tag{1}$$

$$A_{\text{eff}} = \sqrt{A_x^2 \sin^2\theta \cos^2\varphi + A_y^2 \sin^2\theta \sin^2\varphi + A_z^2 \cos^2\theta} \tag{2}$$

for a specific orientation of the nitroxide molecule with respect to the external magnetic field characterized by the polar θ and azimuthal φ angles, respectively. These simplified expressions apply under the assumption that the principal axis systems of the g- and hyperfine tensor coincide. We expect a single-crystal-like spectrum with three peaks for a single orientation, leading to three resonance offsets $\Omega(\theta,\varphi)$. To account for the resonance offset in our density operator formalism simulations of the tunneling ESEEM contribution, we modify the single orientation spin Hamiltonian to

$$\hat{H}(\phi,\theta,\varphi) = \Omega(\theta,\varphi)\hat{S}_z + \omega_I(\hat{I}_{1,x}+\hat{I}_{2,x}+\hat{I}_{3,x}) + A_I(\phi,\theta,\varphi)\hat{S}_z\hat{I}_{1,z} + A_{II}(\phi,\theta,\varphi)\hat{S}_z\hat{I}_{2,z} + A_{III}(\phi,\theta,\varphi)\hat{S}_z\hat{I}_{3,z} \tag{3}$$

$$+ B_I(\phi,\theta,\varphi)\hat{S}_z\hat{I}_{1,x} + B_{II}(\phi,\theta,\varphi)\hat{S}_z\hat{I}_{2,x} + B_{III}(\phi,\theta,\varphi)\hat{S}_z\hat{I}_{3,x} \tag{4}$$

and calculate the secular and pseudo-secular hyperfine coupling constants $A_i(\phi,\theta,\varphi)$ and $B_i(\phi,\theta,\varphi)$, respectively, as described in the main text. To account for off-resonant excitation during pulse application, we modify the pulse Hamiltonian in the density operator simulations to

$$\hat{H}_{\text{pulse}} = t_p\left(\omega_1\hat{S}_{x,y} + \hat{H}_{\text{free}}\right) \tag{5}$$

where t_p is the pulse length, ω_1 is the nutation frequency of the pulse, $\hat{S}_{x,y}$ is the pulse operator acting in either x- or y-direction and $\hat{H}_{\text{free}}$ is the full state Hamiltonian describing the spin system.

We fitted the X- and Q-band spectra of TEIO in cis/trans decalin to obtain the g- and A(^{14}N)-tensor values of $g_x = 2.0091$, $g_y = 2.0061$, $g_z = 2.0023$ and $A_x = 16.1$ MHz, $A_y = 19.7$ MHz, $A_z = 88.0$ MHz (see Fig. S2(a)). Using these tensors, we simulated the tunneling ESEEM

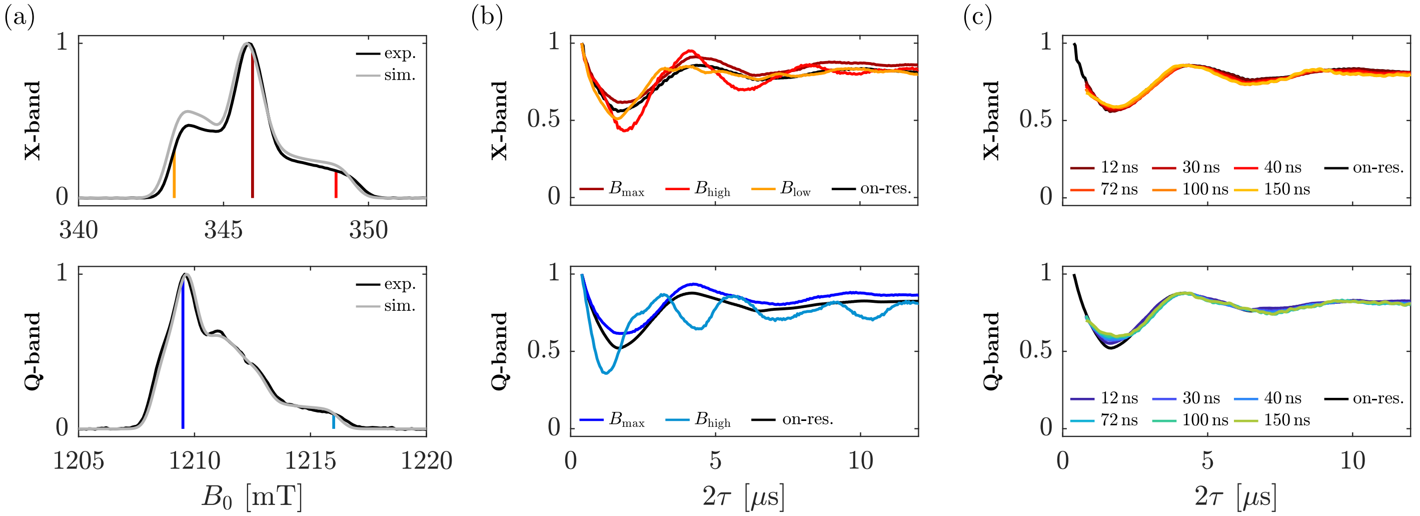

Fig. S2 Influence of selective excitation of the electron spin spectrum on the tunneling ESEEM signal. (a) *top:* Experimental (black) and simulated (gray) spectrum of TEIO in cis/trans decalin at *top:* X-band and *bottom:* Q-band measured at a temperature of 20 K. The colored lines illustrate the magnetic field positions for the tunneling ESEEM simulations. (b) The simulated tunneling ESEEM signals for different field positions at *top:* X-band and *bottom:* X-band are compared with the simulation considering on-resonant excitation of the whole nitroxide spectrum like in the MQR model. (c) Influence of the excitation bandwidth, i.e. pulse length, on the tunneling ESEEM signal at B_{max} for *top:* X-band and *bottom:* Q-band. The indicated pulse length corresponds to the $\pi/2$-pulse in the Hahn echo sequence. The π-pulse length implemented in the simulations is twice as long as the $\pi/2$-pulse.

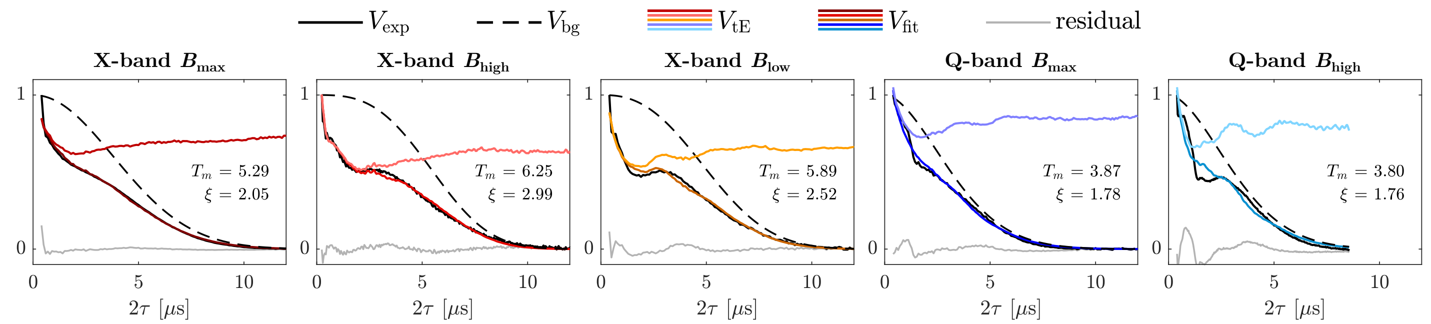

Fig. S3 Experimental tunneling ESEEM of TEIO at different spectral positions and microwave frequencies. The experimental data measured at 20 K is shown as a black line. We fitted the experimental data with a fixed Gaussian rotation barrier distribution with V_{av} =1423 K and σ =168 K. The phase memory time T_m and the stretch parameter ξ were evaluated using least-squares fitting.

contribution for one methyl group ($m = 3$) of the nitroxide TEIO for different frequency bands as well as spectral positions with a fixed rotation barrier $V_3 = 1500$ K, which corresponds to a tunneling frequency $\nu_t = 482$ kHz. We carried out the simulations for 80'401 orientations (201 knots) of the nitroxide with respect to the magnetic field generated by the `sphgrid`-function in EasySpin (version 5.2.35) individually and sum up the single-orientation signals to obtain the overall tunneling ESEEM contribution. The simulations at different spectral positions and microwave frequencies were carried out using $t_{p,\pi/2} = 40$ ns and $t_{p,\pi} = 80$ ns as pulse lengths in the two-pulse ESEEM sequence. The results are presented in Fig.S2(b). Fig. S2(c) additionally shows the influence of the excitation bandwidth on the simulated tunneling ESEEM contribution by considering different pulse lengths.

In Fig. S2(b) we observe that the tunneling ESEEM signal simulated for the field position with maximal signal intensity (B_{max}) has a modulation depth and a modulation period comparable to the simulation, where on-resonant excitation of all orientations with the same weight is assumed. For ESEEM measurements at other field positions (B_{low} and B_{high}), the tunneling ESEEM signal exhibits different modulation depth and period, especially at B_{high}, compared to the simulation for B_{max} and the on-resonance case. Additionally, simulating the tunneling ESEEM contribution including selective excitation requires considering a large number of nitroxide orientations to obtain good signal quality, since the number of sampled orientations represents how well the nitroxide spectrum is sampled in terms of a density of resonance offsets. The excitation bandwidth is directly related to the pulse length - the longer the pulse, the narrower the excitation bandwidth and less spin packets and orientations in the EPR-spectrum are excited. Fig. S2(c) shows that more selective excitation of the EPR-spectrum at B_{max} for longer pulse lengths only weakly influences the tunneling ESEEM contribution. The modulation depth remains almost constant for $\pi/2$-pulse lengths between 12 ns and 72 ns, and starts becoming smaller for pulse lengths of 100 ns and longer, especially at Q-band.

Overall, our simulations suggest that the MQR model presented earlier[2] is applicable for tunneling ESEEM measurements performed at the magnetic field position with maximal spectrum intensity and using short pulses, that excite a substantial fraction of orientations in the spectrum. For the extraction of rotation barrier distributions with the MQR model from ESEEM measurements at field positions that do not correspond to a large variety of orientations, as for example at B_{high}, we would need to account for orientation selection, which would considerably complicate the analysis.

Additionally, we analyzed Hahn echo decay data of TEIO measured at different spectral positions using a tunneling ESEEM kernel considering the resonance offset in the spin Hamiltonian and real pulses. We simulated the kernel considering only 5101 orientations (51 knots), since otherwise computational time becomes unmanageable as well as we run into memory issues. We fitted the matrix-dependent relaxation parameters using ordinary least-squares, while keeping the rotation barrier distribution parameters frozen. We simulated the tunneling ESEEM contributions with V_{av} =1423 K and σ =168 K, which correspond to the extracted distribution parameters for TEIO at 20 K assuming only on-resonant excitation of the whole spectrum in the kernel (see Tab. 2). The results are illustrated in Fig. S3. The experimental data (black signals in Fig. S3) show differences in tunneling ESEEM modulation depth for different spectral positions at both X- and Q-band. The modulation depth is larger at B_{high} and B_{low} at X-band than for the measurement carried out at B_{max}. Also at Q-band the tunneling ESEEM exhibits larger modulation depth at B_{high} than at B_{max}. The simulated tunneling ESEEM contributions also reflect different modulation depths, however they do not agree with the experimental data perfectly. Possibly, the chosen rotation barrier distribution function is too simplified which leads to a deviation of the simulated tunneling contribution. Alternatively, our assumption might be wrong that the rotation barrier distribution, i.e. the local hindrance, is uncorrelated from the resonance offset and therefore also the orientation of the g- and A-tensors. In general, our analysis shows that selective excitation of the EPR-spectrum leads to differences in the observed tunneling ESEEM, which we can only rationalize qualitatively with our simulations.

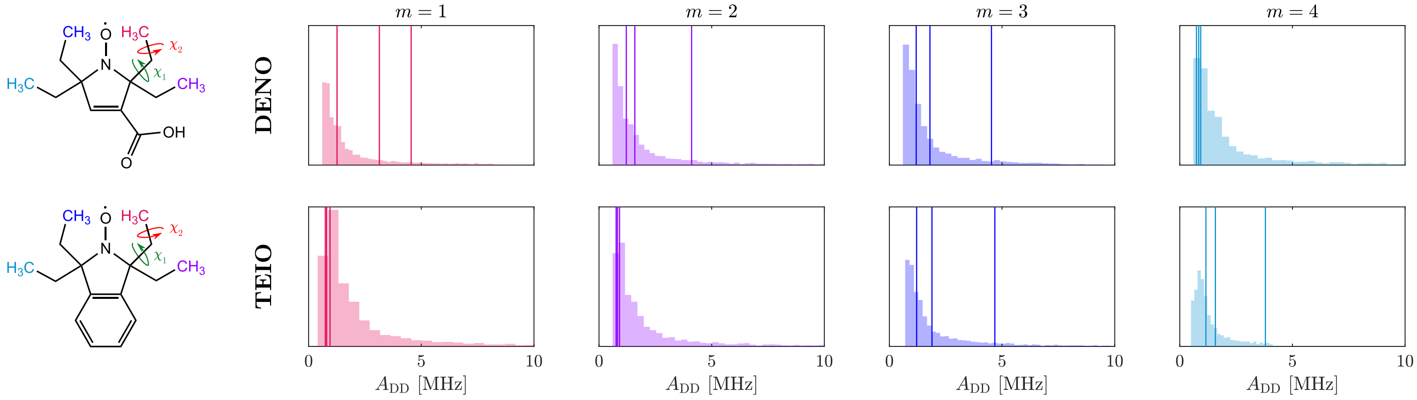

Fig. S4 Conformational flexibility of ethyl groups and resulting dipolar coupling constants. The two dihedral angles χ_1 and χ_2 represent the rotational degrees of freedom of the ethyl substituents and are illustrated in the molecular structures of DENO and TEIO for one ethyl group. The histograms show the resulting distribution of dipolar coupling constants A_{DD} for the evaluated conformations of ethyl rotor m. The solid lines represent the dipolar coupling constants calculated from the geometry-optimized structure, which are implemented in the tunneling ESEEM kernel simulations.

S3 Ethyl group conformers of DENO and TEIO

We examined the dipolar coupling constants between the electron spin and the ethyl group rotor protons for different ethyl group conformers. We used the geometry-optimized structures of DENO and TEIO and fixed all atom positions except the ethyl group (-C_2H_5). We performed subsequent rotation of both dihedral angles χ_1 and χ_2 and saved the respective ethyl group atom positions. From the evaluated atom positions, we calculated the dipolar coupling constant A_{DD} for every conformer using the point-dipole approximation. We approximate the electron spin in the middle of the N-O-bond. Fig. S4 presents the results for DENO and TEIO. All four methy rotors in ethyl groups of DENO and TEIO show similar dipolar coupling constant distributions with the majority of couplings between 500 kHz and 5 MHz. The dipolar coupling constants calculated from the geometry-optimized structure (solid lines in Fig. S4) represent characteristic values of the conformational ensemble of the ethyl groups.

S4 Fitted model parameters for equivalent-rotor nitroxides

We investigated the tunneling behaviour of the equivalent-rotor nitroxides DENO, TEIO, cyc-DOXYL and 2-DOXYL-C_{11} by fitting experimental X-band data measured at B_{max}. Additionally, we compared the extracted MQR model parameters for different temperatures of the methyl rotors in the ethyl-group containing nitroxides DENO and TEIO. The results are presented in Tab. 2.

The phase memory time T_m characterizing the matrix-dependent relaxation process remains almost constant in the temperature range between 10 K and 60 K for DENO. Also the stretch parameter ξ of DENO only changes slightly in the investigated temperature range. This behaviour of the matrix-driven decoherence is characteristic for the temperature range where nuclear spin diffusion (NDS) is the dominant process causing spin-spin relaxation[3,4]. For TEIO, both T_m and ξ increase significantly from 20 K to 40 K. However, these parameters cannot be compared directly since we measured the 20 K experimental data with longer pulses. This results in a smaller excitation bandwidth, which influences the decoherence behaviour because different spin packets in the spectrum are excited

Table 2 Evaluated model parameters for equivalent-rotor nitroxides at different temperatures. The table indicates the methyl rotor containing alkyl group in the respective nitroxide and the hyperfine (HF) approximation used in the tunneling ESEEM kernel to fit the experimental X-band data measured at B_{max}. The matrix-driven decoherence process is characterized by the phase memory time T_m and the stretch parameter ξ. The tunneling ESEEM contribution is described by a Gaussian rotation barrier distribution parameterized by the mean V_{av} and width σ.

nitroxide	T [K]	alkyl group	HF-approx.	T_m [μs]	ξ	V_{av} [K]	σ [K]
DENO	10 K	ethyl	A_{DD} secular	4.48	2.25	1404	155
	20 K	ethyl	A_{DD} secular	4.57	2.39	1411	152
	40 K	ethyl	A_{DD} secular	4.56	2.38	1418	157
	60 K	ethyl	A_{DD} secular	4.39	2.32	1451	175
TEIO	20 K*	ethyl	A_{DD} secular	5.32	2.08	1423	168
	40 K	ethyl	A_{DD} secular	5.92	2.49	1392	249
cyc-DOXYL	20 K	methyl	A_{FC} + A_{DD} secular	4.69	2.57	1630	163
2-DOXYL-C_{11}	20 K	methyl	A_{FC} + A_{DD} secular	4.69	2.64	1607	147

* This measurement was carried out with $t_{\pi/2} = 60$ ns and $t_\pi = 120$ ns.

and detected by the pulse sequence[5]. However, our simulations in Section S2 showed that for measurements at $B_{\max}$, the pulse length has no significant impact on the tunneling ESEEM contribution, which allows us to compare the distribution parameters for DENO and TEIO.

Comparing the rotation barrier distribution parameters V_{av} and σ for DENO, we observe that the distribution mean remains almost constant between 10 K and 40 K and shifts to slightly higher values for 60 K. The distribution width indicates that the distribution becomes broader for higher temperatures. For TEIO we observe that V_{av} is about 30 K higher at a temperature of 20 K than for 40 K, but the extracted distribution width is higher for the 40 K-measurement. The observed trends might suggest that for higher temperatures, when more free volume becomes available in the glass, the ethyl groups experience more conformational flexibility resulting in broader rotation barrier distributions. Generally, the temperature dependence of the rotation barrier distribution parameters are different than in our previous study on the methyl rotors of H-mNOHex in OTP[2], where we observed a decrease in distribution width in the temperature range between 10 K - 60 K. However, it is difficult to directly compare the temperature behaviour of H-mNOHex with the DENO and TEIO investigated here, since the nitroxide backbone structures as well as the matrices are different and their influence on the tunneling ESEEM contribution is not clear yet.

S5 Impact of classical rotation on T_m for methyl and ethyl-containing nitroxides

When the rate of classical rotation of a methyl group is comparable to the anisotropy in hyperfine couplings of a paramagnetic center to the inequivalent methyl protons $1/T_m$ increases[3], which has been observed for nitroxide radicals[6,7] and Cr(V)-complexes[7]. The temperature range in which the maximum enhancement in $1/T_m$ is observed increases as the barrier to rotation increases. For methyl-containing nitroxide radicals this process dominates $1/T_m$ at temperature above about 80 K. The temperature dependence of $1/T_m$ for the ethyl-containing nitroxides DENO and TEIO in 1:1 decalin:OTP are compared with values for geminal methyl-containing nitroxides MTSL in 1:1 water:glycerol and TMIO in 1:1 decalin:OTP in Figure S5. OTP is ortho-terphenyl. Although the tunneling ESEEM causes uncertainty in the values of $1/T_m$ obtained by fitting to the data, a simple exponential fit was employed to provide a systematic approach to characterizing the temperature dependence of the decays. The maximum in $1/T_m$ occurs at about 150 K for TMIO and MTSL. The maximum in $1/T_m$ is at about 110 K for TEIO and about 120 K for DENO, which is significantly lower than for TMIO or MTSL. This temperature-dependent analysis of classical methyl rotation indicates that the rotation barrier of the methyl rotors in ethyl groups is lower than for the geminal methyl groups. Analysis of the temperature dependence of $1/T_m$ also shows that the rotation barrier for methyl groups attached to a chelate ring is higher than for methyl group of an ethyl attached to a chelate ring in Cr(V)-complexes[7]. These observations are consistent with the conclusion from the analysis of the tunneling ESEEM data described in the main text that the rotation barrier is higher for methyl group rotors than for the methyl rotors of an ethyl group.

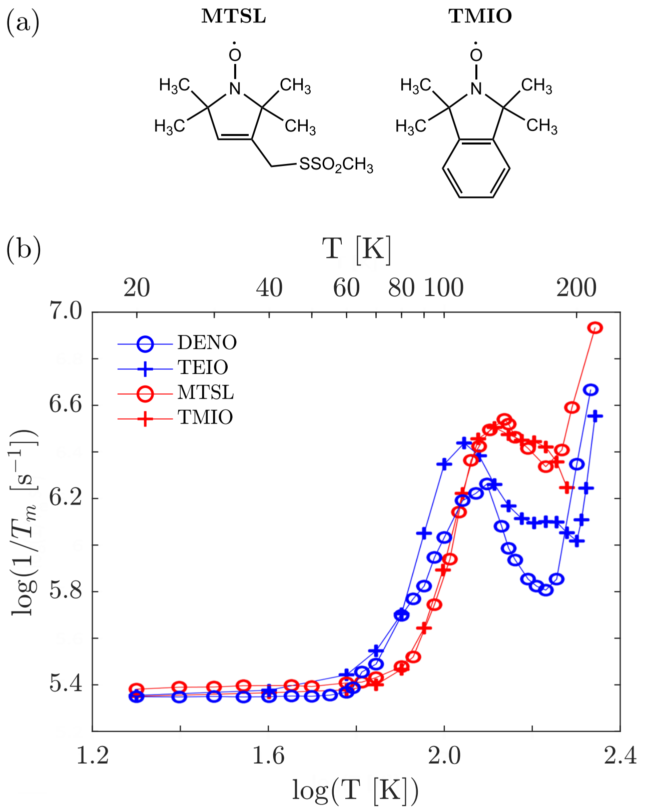

Fig. S5 Comparison of classical barrier of rotation of methyl and ethyl group containing nitroxides. (a) The molecular structures of the methyl group containing nitroxides MTSL and TMIO are shown. (b) Temperature dependence of $1/T_m$ for DENO (blue circles), TEIO (blue plus), MTSL (red circles), and TMIO (red plus).

S6 Comparison of Gaussian and non-parametric $P(V_3)$ for equivalent-rotor nitroxides

The rotation barrier distribution can be represented by a parametric model, for example a Gaussian distribution, or a non-parametric model. Using a parametric model imposes a certain bias on the shape and symmetry of the rotation barrier distribution, which might hide details of the underlying $P(V_3)$ or even corrupt the analysis and interpretation of our experimental findings. Using a non-parametric vector to describe the rotation barrier distribution requires regularization methods to stabilize the solution. In our case, we applied a non-negativity constraint together with Tikhonov regularization and a compactness penalty to extract the non-parametric rotation barrier distribution via regularized least-squares

$$P_{\mathrm{fit}} = \underset{P \geq 0}{\operatorname{argmin}} \left\{ \| \boldsymbol{V}_{\exp} - \mathbf{K}\boldsymbol{P} \|^2 + \alpha^2 \|\mathbf{L}\boldsymbol{P}\| + \beta^2 \sigma^2(\boldsymbol{P}) \right\}. \tag{6}$$

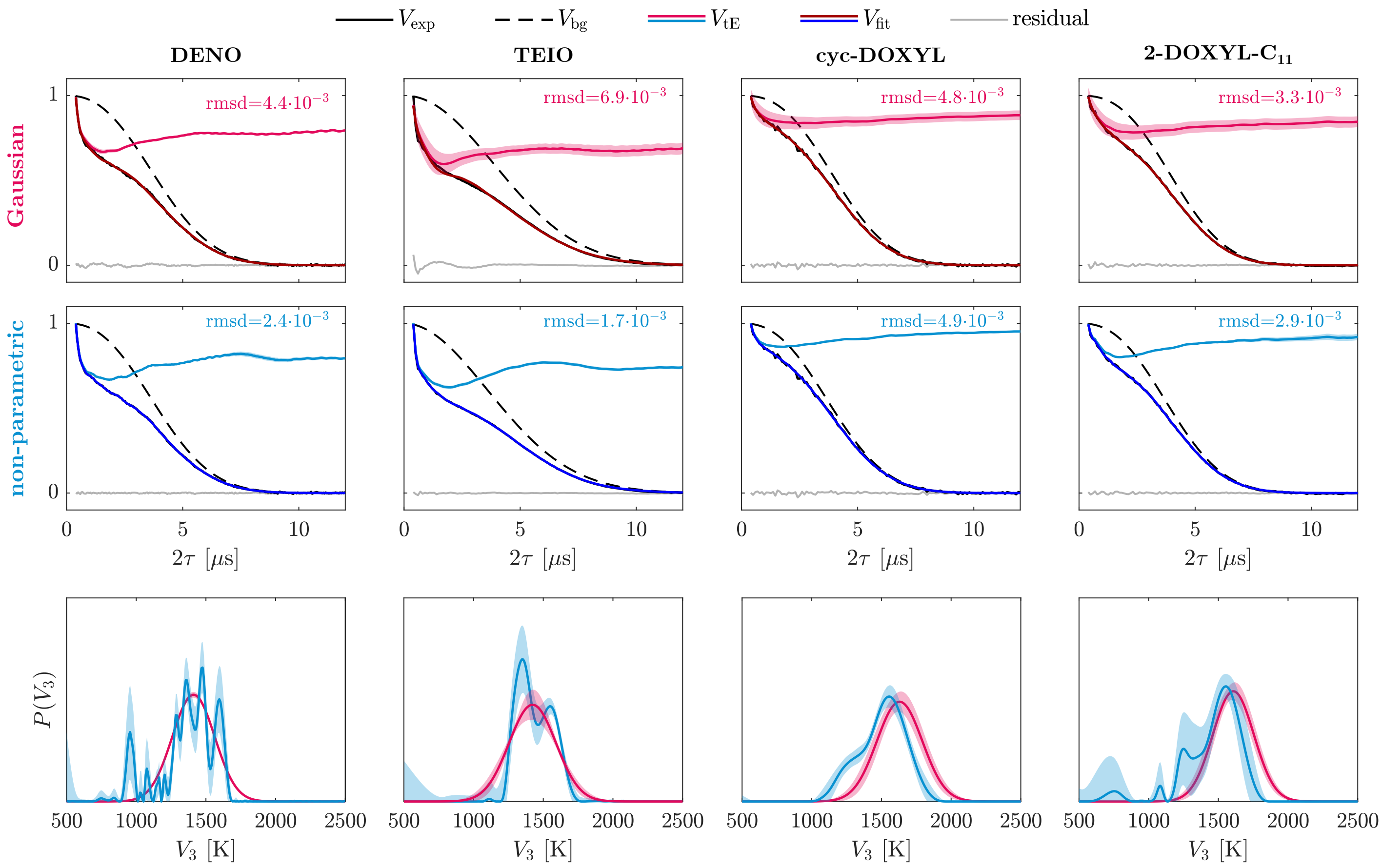

Fig. S6 Comparison of inferred rotation barrier distribution of equivalent-rotor nitroxides using a Gaussian (red, top) and a non-parametric (blue, middle) model for the distribution. The root-mean-square deviation (rmsd) is provided for the time-domain fits to assess the goodness-of-fit. The shaded areas represent 95 % covariance-based confidence intervals.

The first term assures agreement of the model prediction with the experimental data, whereas the second term introduces Tikhonov regularization by biasing the solution to smoothness of the inferred distribution using the differential operator **L**. The third term imposes compactness on the rotation barrier distribution by introducing a penalty for larger distribution variance $\sigma^2(P)$. This suppresses unreasonable distribution mass at large values of the rotation barrier. The optimal regularization parameter α is found using the normalized cumulative periodogram (NCP) [8] within a range of $\alpha = [1\times10^4, 5\times10^6]$. The optimal value for the compactness parameter β is evaluated using the information complexity criterion (ICC) [9] implemented as a default in DeerLab [10]. This fitting procedure using a non-parametric model was already established in our previous work [2] and is transferable to the current study.

Fig. S6 illustrates the fitting results using a Gaussian (red) and a non-parametric (blue) model for the rotation barrier distribution when analyzing the experimental X-band data recorded at B_{max} and 20 K for DENO, TEIO, cyc-DOXYL and 2-DOXYL-C$_{11}$. We observe that the overall fit quality becomes better according to the root-mean-square deviation (rmsd) when implementing a non-parametric model for the rotation barrier distribution for DENO, TEIO and 2-DOXYL-C$_{11}$. For cyc-DOXYL the rmsd is almost the same independent of the model applied to the rotation barrier distribution, however the goodness-of-fit is excellent in both cases. For all nitroxides the confidence intervals of the tunneling ESEEM contribution become smaller and the fit residuals become almost flat when using a non-parametric distribution model. Moreover, the inferred non-parametric rotation barrier distributions show significant overlap with the respective Gaussian distributions. The Gaussian distributions of cyc-DOXYL and 2-DOXYL-C$_{11}$ are shifted to slightly higher rotation barriers compared to their non-parametric fits. For all nitroxides, the Gaussian rotation barrier distribution is slightly broader than the non-parametric distribution. Overall, these results indicate that irrespective of the model for the rotation barrier distribution, the MQR model yields fitting results with the same fundamental rotation barriers that are necessary to represent the observed tunneling ESEEM accurately. Moreover, these results suggest when analyzing the experimental tunneling ESEEM with the MQR model, we are able to detect differences in the rotation barrier distributions of geminal methyl group rotors and methyl rotors in ethyl groups independent of the model for the distribution. This further underlines that the tunneling ESEEM contribution is sensitive to the local environments of the investigated rotors and our model can quantitatively describe the observed tunneling behaviour.

S7 Fitted model parameters for mixed-rotor nitroxides using different distribution models

To investigate the mixed-rotor nitroxides' tunneling behaviour, we used a combination of different fitting approaches to identify which rotor type is responsible for the experimentally observed the tunneling ESEEM. Implementing a bivariate distribution in the MQR model requires Monte-Carlo sampling of the parameter space while monitoring the objective function to evaluate a fitting solution. We implemented two different boundary conditions on the distribution means $V_{i,\mathrm{av}}$ of rotor type i, which we refer to as the unconstrained and constrained MC fitting procedure. For the unconstrained fitting, we employed lower and upper bounds for $V_{i,\mathrm{av}}$ of 1200 K and 2000 K for both rotor types. In the constrained fitting approach, we ensure that the rotation barrier distribution for geminal methyl group rotors has a higher mean value than the one for methyl rotors in longer alkyl groups. Therefore, the boundaries for $V_{1,\mathrm{av}}$ are 1500 K - 2000 K, while for $V_{2,\mathrm{av}}$ we implemented 1200 K - 1500 K. Additionally, we only considered one rotor type in the fitting procedure and therefore a univariate rotation barrier distribution was employed in the MQR model. Tab. 3 lists the examined results from these three fitting approaches. We refer to the main text for discussion of the results.

Table 3 Evaluated fit parameters using different distribution models for the mixed-rotor nitroxides. The phase memory time T_m and the stretch parameter ξ describe the matrix-dependent relaxation contribution considered in the MQR model. Fitting a bivariate rotation barrier distribution model was carried out by Monte-Carlo (MC) sampling of the parameter space using 50'000 samples. For the unconstrained fitting (uncon.), we allowed values between 1200 K - 2000 K for both rotation barrier distribution means $V_{i,\mathrm{av}}$, whereas for the constrained fitting (con.), the geminal methyl group rotor distribution mean $V_{1,\mathrm{av}}$ was constrained between 1500 K - 1800 K and the distribution mean for methyl rotors in the ethyl, propyl, butyl and pentyl groups $V_{2,\mathrm{av}}$ could only take values between 1200 K and 1500 K. The univariate distribution model only considers one rotor type in the MQR model. The root-mean-square-deviation (rmsd) serves as a fit quality metric.

nitroxide	$P(V_3)$	rotor	T_m [μs]	ξ	$V_{i,\mathrm{av}}$ [K]	σ_i [K]	rmsd [10^{-3}]
3-DOXYL-C_5	bivariate uncon.	methyl<br>ethyl	4.62	2.35	1522<br>1902	171<br>228	4.4
	bivariate con.	methyl<br>ethyl	4.19	2.12	1614<br>1379	209<br>195	5.1
	univariate	methyl<br>ethyl	4.24<br>4.10	2.14<br>2.04	1580<br>1411	109<br>171	4.7<br>5.8
3-DOXYL-C_{14}	bivariate uncon.	methyl<br>ethyl	4.63	2.63	1602<br>1868	97<br>192	4.6
	bivariate con.	methyl<br>ethyl	4.43	2.45	1594<br>1331	77<br>250	4.6
	univariate	methyl<br>ethyl	4.51<br>4.52	2.52<br>2.55	1597<br>1607	88<br>76	3.7<br>4.6
4-DOXYL-C_{14}*	bivariate uncon.	methyl<br>propyl	4.56	2.50	1643<br>1386	62<br>118	2.8
	bivariate con.	methyl<br>propyl	4.48	2.35	1608<br>1349	107<br>157	3.1
	univariate	methyl<br>propyl	4.34<br>4.19	2.27<br>2.07	1553<br>1447	67<br>107	3.4<br>4.3
5-DOXYL-C_{14}	bivariate uncon.	methyl<br>butyl	4.48	2.15	1601<br>1879	74<br>142	3.8
	bivariate con.	methyl<br>butyl	4.24	2.15	1607<br>1306	80<br>80	3.9
	univariate	methyl<br>butyl	4.33<br>4.06	2.26<br>2.00	1607<br>1535	63<br>50	3.5<br>11.6
6-DOXYL-C_{14}	bivariate uncon.	methyl<br>pentyl	4.55	2.51	1626<br>1430	117<br>230	2.9
	bivariate con.	methyl<br>pentyl	4.53	2.58	1622<br>1310	122<br>212	2.9
	univariate	methyl<br>pentyl	4.44<br>4.22	2.43<br>2.09	1615<br>1490	126<br>50	2.6<br>8.8

* This measurement was carried out with $t_{\pi/2}$ = 80 ns and t_{π} = 160 ns.

Notes and references

1 Schrödinger, LLC, *The PyMOL Molecular Graphics System, Version 2.5.2*, 2021.

2 A. Eggeling, J. Soetbeer, L. Fábregas-Ibáñez, D. Klose and G. Jeschke, *Physical Chemistry Chemical Physics*, 2023, **25**, 11145–11157.

3 S. S. Eaton and G. R. Eaton, *Distance Measurements in Biological Systems by EPR*, Springer US, Boston, MA, 2000, pp. 29–154.

4 S. S. Eaton and G. R. Eaton, *eMagRes*, Wiley, 2016, pp. 1543–1556.

5 R. Husted, J.-L. Du, G. R. Eaton and S. S. Eaton, *Magnetic Resonance in Chemistry*, 1995, **33**, S66–S69.

6 S. A. Dzuba, E. S. Salnikov and L. V. Kulik, *Applied Magnetic Resonance*, 2006, **30**, 637–650.

7 K. Nakagawa, M. B. Candelaria, W. W. C. Chik, S. S. Eaton and G. R. Eaton, *Journal of Magnetic Resonance (1969)*, 1992, **98**, 81–91.

8 P. C. Hansen, M. E. Kilmer and R. H. Kjeldsen, *BIT Numerical Mathematics*, 2006, **46**, 41–59.

9 J. M. Ferguson, M. L. Taper, R. Zenil-Ferguson, M. Jasieniuk and B. D. Maxwell, *Frontiers in Ecology and Evolution*, 2019, **7**, 1–15.

10 L. Fábregas Ibáñez, G. Jeschke and S. Stoll, *Magnetic Resonance*, 2020, **1**, 209–224.
